# Single‐cell gene regulatory network analysis for mixed cell populations

**DOI:** 10.1002/qub2.64

**Published:** 2024-07-02

**Authors:** Junjie Tang, Changhu Wang, Feiyi Xiao, Ruibin Xi

**Affiliations:** ^1^ School of Mathematical Sciences and Center of Statistical Science Peking University Beijing China; ^2^ Academy for Advanced Interdisciplinary Studies Peking University Beijing China

**Keywords:** gene regulatory network, graphical model, precision matrix, variational inference, single‐cell RNA sequencing

## Abstract

Gene regulatory network (GRN) refers to the complex network formed by regulatory interactions between genes in living cells. In this paper, we consider inferring GRNs in single cells based on single‐cell RNA sequencing (scRNA‐seq) data. In scRNA‐seq, single cells are often profiled from mixed populations, and their cell identities are unknown. A common practice for single‐cell GRN analysis is to first cluster the cells and infer GRNs for every cluster separately. However, this two‐step procedure ignores uncertainty in the clustering step and thus could lead to inaccurate estimation of the networks. Here, we consider the mixture Poisson log‐normal model (MPLN) for network inference of count data from mixed populations. The precision matrices of the MPLN are the GRNs of different cell types. To avoid the intractable optimization of the MPLN’s log‐likelihood, we develop an algorithm called variational mixture Poisson log‐normal (VMPLN) to jointly estimate the GRNs of different cell types based on the variational inference method. We compare VMPLN with state‐of‐the‐art single‐cell regulatory network inference methods. Comprehensive simulation shows that VMPLN achieves better performance, especially in scenarios where different cell types have a high mixing degree. Benchmarking on real scRNA‐seq data also demonstrates that VMPLN can provide more accurate network estimation in most cases. Finally, we apply VMPLN to a large scRNA‐seq dataset from patients infected with severe acute respiratory syndrome coronavirus 2 (SARS‐CoV‐2) and find that VMPLN identifies critical differences in regulatory networks in immune cells between patients with moderate and severe symptoms. The source codes are available on the GitHub website (github.com/XiDsLab/SCVMPLN).

## 1 INTRODUCTION

Gene regulatory network (GRN), which represents the regulatory relationships between genes, is important for understanding the complex biological system [1]. GRNs can be inferred based on gene expression data such as RNA sequencing (RNA‐seq) data. Bulk expression data are most commonly used for GRN inference, and numerous methods have been developed (see Refs [2, 3] and references therein). However, bulk data are profiled from pooled cell populations and thus can only provide average expressions of many cells. The recent development of single‐cell RNA‐sequencing (scRNA‐seq) technologies can measure gene expression at the single‐cell level [4, 5], thus offering an unprecedented opportunity for single‐cell GRN inference.

**FIGURE 1:**
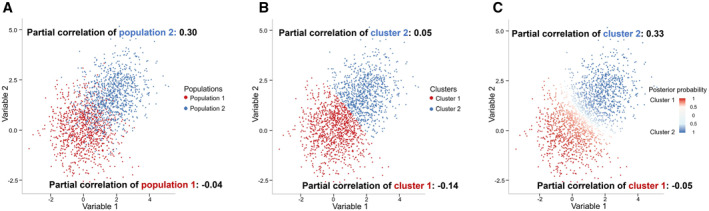
An example of data from a two‐dimensional mixture Gaussian distribution. We sample 2000 observations from the Gaussian mixture model $0.5\;N\left(\mu =\left(0,0\right),\Sigma ={\Theta_{1}}^{-1}\right)+0.5\;N\left(\mu =\left(1.7,1.7\right),\Sigma ={\Theta_{2}}^{-1}\right)$. The diagonal elements of precision matrices *Θ*_1_ and *Θ*_2_ are 1, and the non‐diagonal elements of *Θ*_1_ and *Θ*_2_ are 0 and −0.3, respectively. (A) The scatter plot of the 2000 samples with true labels (red: population 1, blue: population 2). Using the true label, as expected, samples in population 1 demonstrate a partial correlation very close to 0, and samples in population 2 have a large nonzero partial correlation (0.30). (B) The samples are first clustered using the K‐means algorithm. Partial correlations are calculated for each cluster and are far away from the true partial correlations, which would lead to incorrect network estimates. (C) Parameters are estimated by maximizing the likelihood of the mixture Gaussian distribution. The estimates of the partial correlations are now close to the true values.

To account for the unique features of scRNA‐seq data, several GRN inference methods based on scRNA‐seq data have been developed [6, 7, 8]. These methods often make the implicit assumption that all cells share the same GRN. However, single cells in scRNA‐seq data usually belong to multiple cell types, and each cell type has its own specific GRN and expression pattern. The cell identities are unknown and have to be determined using scRNA‐seq data. To infer GRNs of different cell types, one has to first assign single cells to different cell types (e.g., by clustering) and then estimate the GRNs using available methods. This two‐step procedure can provide accurate GRN estimation if different cell types are well separated. If, instead, different cell types have a higher mixing degree, a large proportion of cells cannot be confidently assigned to a cell type, and the ambiguity of the cell type assignment could seriously influence the performance of GRN inference (see Figure 1A,B for an inspiring simulation example).

Here, we consider developing a GRN inference method based on scRNA‐seq data with mixed cell populations using mixture models. One major advantage of mixture models is that cell types need not be predetermined before GRN inference. Instead, mixture models can allow joint analyses of clustering and GRN inference and thus could give better GRN estimation when different cell types are poorly separated. Since scRNA‐seq data are count data, often rather small count data with many zeros (i.e., the high dropout problem), graphical models for count data would be more suitable than the widely used Gaussian graphical model (GGM) [9, 10] or its mixture version. Available graphical models for count data include generalized linear models [11], Poisson graphical models (PGMs) [12, 13], and Poisson log‐normal (PLN) models [14, 15, 16, 17]. Compared with PGMs, the PLN models can model the over‐dispersion commonly observed in scRNA‐seq data [18]. Therefore, we propose to use the mixture PLN (MPLN) model for GRN inference in single cells.

A non‐negative integer random vector $Y={\left(Y_{1},\text{…},Y_{p}\right)}^{T}\in R^{p}$ follows a PLN distribution, if conditional on a latent random vector $X={\left(X_{1},\text{…},X_{p}\right)}^{T}\in R^{p}$ with **X** ∼ *N*(μ,Σ), each element of **Y** independently follows the univariate Poisson distribution, that is, *Y*_*j*_ ∼ Poisson(exp(*X*_*j*_)) (*j* = 1,…,*p*). Similar to the GGM, the network of the PLN model is the precision matrix Θ = Σ^−1^ of the latent variable **X**. The MPLN model is a mixture of different PLN models. The precision matrix of each component of the MPLN model represents the network of a cell type. Directly maximizing the lasso‐penalized log‐likelihood of the MPLN is computationally intractable. We adopt the variational inference approach [19, 20] and develop an algorithm called variational mixture Poisson log‐normal (VMPLN) for simultaneous analyses of clustering and network inference. We compare VMPLN with popular graphical methods and state‐of‐the‐art single‐cell regulatory network inference methods. Comprehensive simulation shows that VMPLN achieves better performance, especially in scenarios where different cell types have a high mixing degree. Benchmarking on real scRNA‐seq data also demonstrates that VMPLN can provide more accurate network estimation in most cases. Finally, we apply VMPLN to a large scRNA‐seq dataset from patients infected with severe acute respiratory syndrome coronavirus 2 (SARS‐CoV‐2) and find that VMPLN identifies critical differences in regulatory networks in immune cells between patients with moderate and severe symptoms.

## 2 RESULTS

### 2.1 The framework of VMPLN

To infer the GRN of mixed populations in scRNA‐seq data, we model scRNA‐seq count expression data from mixed populations using the mixture Poisson log‐normal (MPLN) model (See Section 2.1.1) and propose an efficient algorithm based on the variational inference called VMPLN to estimate the GRNs of different cell populations (See Section 2.1.2).

#### 2.1.1 The MPLN of scRNA‐seq data from mixed populations

Suppose that a scRNA‐seq dataset consists of *n* cells and *p* genes. Let $Y_{i}={\left(Y_{i1},\text{…},Y_{ip}\right)}^{T}$ be the observed expression vector of the *i*th cell, where *Y*_*ij*_s are all non‐negative integers. Single cells in scRNA‐seq data belong to G different cell types, and each cell type has its own unique mean gene expression and regulatory network. The cell identities are unknown and have to be determined based on the observed data **Y**. The observed expression *Y*_*ij*_ is a noisy measurement of the true expression exp(*X*_*ij*_) of the *i* th cell at the *j* th gene. Conditional on *X*_*ij*_, we assume that *Y*_*ij*_ follows a Poisson distribution with a mean *λ*_*ij*_ = *l*_*i*_ exp(*X*_*ij*_), where *l*_*i*_ is the library size of the *i* th cell and can be readily estimated using available methods [21, 22]. Denote $X_{i}={\left(X_{i1},\text{…},X_{ip}\right)}^{T}$ be the logarithm of the true expression vector of the *i* th cell. We assume that the logarithm of true expressions of single cells in the *g* th cell type are normally distributed with a mean μ_*g*_ and a covariance ${\Theta_{g}}^{-1}$. Thus, given the underlying cell type *Z*_*i*_ = *g* (*g* = 1,…,*G*) of the *i* th cell, the conditional distribution of **X**_*i*_ is $N\left(\mu_{g},{\Theta_{g}}^{-1}\right)$. The precision matrices Θ_*g*_ represent the population‐specific GRN. We further assume that *Z*_*i*_ follows a multinomial distribution Multinomial (1,*π*) where *π*=(*π*_1_,…,*π*_*G*_) is the proportion parameter representing the composition of cell types. In summary, we have the following MPLN model for scRNA‐seq data from mixed populations:

(1) $$\begin{array}{c} Y_{i}\;|\;X_{i}\;∼\;\prod_{j=1}^{p}\text{Poisson}\left[l_{i}\;\exp\left(X_{ij}\right)\right],\\ X_{i}\;|\;Z_{i}=g\;∼\;N\left(\mu_{g},{\Theta_{g}}^{-1}\right),\Theta_{g}≻0,\\ Z_{i}∼\text{Multinomial}\left(1,\pi \right), \end{array}$$

where Θ_*g*_ ≻ 0 means that Θ_*g*_ is positive definite.

Let $\theta =(\;\pi ,\mu ={\left\{\mu_{g}\right\}}_{g=1}^{G},\Theta ={\left\{\Theta_{g}\right\}}_{g=1}^{G})$ is denoted as unknown model parameters of the model (1). Let $p\left(Y_{i}\;|\;X_{i}\right)=\prod_{j=1}^{p}\left\{{\left[l_{i}\;\exp\left(X_{ij}\right)\right]}^{Y_{ij}}\exp\left[-l_{i}\;\exp\left(X_{ij}\right)\right]{\left(Y_{ij}!\right)}^{-1}\right\}$ be the conditional probability mass function of **Y**_*i*_ given **X**_*i*_. Suppose that *p*(**X**; *μ*_*g*_, Θ_*g*_) is the density function of the normal distribution with mean *μ*_*g*_ and covariance ${\Theta_{g}}^{-1}$. The conditional density function *p*(**X**_*i*_|*Z*_*i*_; *μ*, Θ) of **X**_*i*_ given *Z*_*i*_ is $p\left(X_{i}\;|Z_{i};\;\mu ,\Theta \right)=\prod_{g=1}^{G}{p\left(X_{i}\;|Z_{i}=g;\;\mu_{g},\Theta_{g}\right)}^{I\left(Z_{i}=g\right)}$. Denote $p\left(Z_{i};\pi \right)=\prod_{g=1}^{G}{\pi_{g}}^{I\left(Z_{i}=g\right)}$ as the probability mass function of the multinomial distribution. The log‐likelihood of the MPLN model (1) is as follows.

(2) $$\begin{array}{c} l_{n}\left(\theta \right)=\sum_{i=1}^{n}\log\left\{p\left(Y_{i};\theta \right)\right\}, \end{array}$$

where *p*(**Y**_*i*_; *θ*) = ∬*p*(**Y**_*i*_|**X**_*i*_) *p*(**X**_*i*_|*Z*_*i*_; μ, Θ) *p*(*Z*_*i*_; *π*)*d* **X**_*i*_ *dZ*_*i*_ is the marginal probability mass function of **Y**_*i*_. Let Θ_*g*,*lm*_ be the (*l*, *m*)th element of Θ_*g*_. The networks are sparse and can be estimated by minimizing the lasso‐penalized negative log‐likelihood:

(3) $$\begin{array}{c} -\frac{1}{n}l_{n}\left(\theta \right)+\lambda_{n}\sum_{g=1}^{G}{\left\|\Theta_{g}\right\|}_{1,\text{off}}, \end{array}$$

where *λ*_*n*_ > 0 is a tuning parameter to control the sparsity of networks and ${\left\|\Theta_{g}\right\|}_{1,\text{off}}=\sum_{l\neq m}\left|\Theta_{g,lm}\right|$ is the off‐diagonal *l*_1_‐norm of Θ_*g*_.

#### 2.1.2 Variational inference for the MPLN

The log‐likelihood Eq. (2) of the MPLN model involves an intractable integration and thus, directly minimizing Eq. (3) is computationally very difficult. We therefore adopt the variational inference approach to estimate the networks [19, 20]. Specifically, we approximate the log‐likelihood Eq. (2) by the evidence low bound (ELBO) $l_{E}\left(\theta ,\eta \right)=E_{q\left(X,Z;\eta \right)}\left\{\log\;p\left(X,Z,Y;\theta \right)-\;\log\;q\left(X,Z;\eta \right)\right\}$, and estimate the model parameters *θ* by minimizing ${-l}_{E}\left(\theta ,\eta \right)+\lambda_{n}\sum_{g=1}^{G}{\left\|\Theta_{g}\right\|}_{1,\text{off}}$, where *p*(**X**, **Z**, **Y**; *θ*) is the complete likelihood of the MPLN model and *q*(**X**, **Z**; *η*) is the variational distribution for latent variables **X** and **Z** with variational parameters *η*.

For computational considerations, we consider the following variational distribution family. Specifically, conditional on *Z*_*i*_ = *g*, this variational distribution family assumes that *X*_*ij*_ (*j* = 1,…,*p*) are independent normal variables with a mean *M*_*g*,*ij*_ and a variance *S*_*g*,*ij*_. The distribution of *Z*_*i*_ is a multinomial distribution with proportion parameters $P_{i}={\left(P_{i1},\text{…},P_{iG}\right)}^{T}$. Denote $M_{g}={\left(M_{g,ij}\right)}_{n\times p}$, $S_{g}={\left(S_{g,ij}\right)}_{n\times p}$, and $P={\left(P_{1},\text{…},P_{n}\right)}^{T}$. The variational parameters are $\eta ={\left\{\eta_{g}\right\}}_{g=1}^{G}={\left\{M_{g},S_{g},{\left\{P_{iG}\right\}}_{i=1}^{n}\right\}}_{g=1}^{G}$ with $\eta \in H=\left\{\eta |\;S_{g,ij}>0,P_{iG}\geq 0,\sum_{g=1}^{G}P_{iG}=1\right\}$. Thus, the variational distribution family that we consider is:

$$L=\left\{q\left(X,Z;\eta \right)|q\left(X,Z;\eta \right)=\prod_{i=1}^{n}\left\{q\left(Z_{i};P_{i}\right)\times \prod_{j=1}^{p}\prod_{g=1}^{G}q\left(X_{ij}|Z_{i}=g;\;M_{g,ij},S_{g,ij}\right)\right\},\eta \in H\;\right\},$$

where *q*(*Z*_*i*_;**P**_*i*_) is the density function of Multinomial (1,**P**_*i*_) and *q*(*X*_*ij*_|*Z*_*i*_ = *g*; *M*_*g*,*ij*_, *S*_*g*,*ij*_) is the density function of *N*(*M*_*g*,*ij*_, *S*_*g*,*ij*_).

Given the two matrices **A** and **B**, **A**⊙**B** is denoted as the Hadamard product, **A**_*i*⋅_ and **A**_⋅*j*_ as the *i* th row, and the *j* th column as the vectors of **A**, respectively. Given a vector **c**, define *D*(**c**) as the diagonal matrix whose diagonal elements are **c**. Denote *θ*_*g*_ = (*π*_*g*_, μ_*g*_, Θ_*g*_) as the unknown model parameters of the *g* th population, and

$$l={\left(l_{1},\text{…},l_{n}\right)}^{T},$$

$$\Sigma_{g,i}=\left(M_{g,i\cdot }-\;\mu_{g}\right){\left(M_{g,i\cdot }-\;\mu_{g}\right)}^{T}+D\left(S_{g,i\cdot }\right),$$

$$F_{1}\left(l_{i},M_{g,ij},S_{g,ij}\right)=\exp\left(M_{g,ij}+\frac{1}{2}S_{g,ij}+\log\;l_{i}\right),$$

$$F_{2}\left(\Theta_{g},M_{g,i\cdot },S_{g,i\cdot },\mu_{g}\right)=\frac{1}{2}\left\{\log\;\det\;\Theta_{g}-\text{tr}\left(\Theta_{g}\Sigma_{g,i}\right)\right\}.$$

With the variational distribution family L, the ELBO can be written as $l_{E}\left(\theta ,\eta \right)=\sum_{g=1}^{G}l_{E}^{\left(g\right)}\left(\theta_{g},\eta_{g}\right)$ with

$$l_{E}^{\left(g\right)}\left(\theta_{g},\eta_{g}\right)=P_{\cdot g}^{T}\left(\Lambda_{g}^{\left(1\right)}-\;\Lambda_{g}^{\left(2\right)}+\Lambda_{g}^{\left(3\right)}\right)\;1_{p}+P_{\cdot g}^{T}\left(\Lambda_{g}^{\left(4\right)}+\Lambda_{g}^{\left(5\right)}\right)+K_{g}\left(Y\right),$$

where

$$K_{g}\left(Y\right)=\sum_{i,j}P_{ig}\left\{-\log\left(Y_{ij}!\right)+Y_{ij}\;\log{\;l}_{i}\right\},$$

$$\Lambda_{g}^{\left(1\right)}=Y⊙M_{g},$$

$$\Lambda_{g}^{\left(2\right)}={\left(\Lambda_{g,ij}^{\left(2\right)}\right)}_{n\times p}={\left(F_{1}\left(l_{i},M_{g,ij},S_{g,ij}\right)\right)}_{n\times p},$$

$$\Lambda_{g}^{\left(3\right)}=\frac{1}{2}\log\;S_{g},$$

$$\Lambda_{g}^{\left(4\right)}=\log\left(\pi_{g}\right)1_{n}-\;\log\left(P_{\cdot g}\right),$$

$$\Lambda_{g}^{\left(5\right)}={\left(\Lambda_{g,i}^{\left(5\right)}\right)}_{n}={\left(F_{2}\left(\Theta_{g},M_{g,i\cdot },S_{g,i\cdot },\mu_{g}\right)\right)}_{n}.$$

In real applications, we may have prior knowledge that some node pairs cannot have direct interactions. In this case, we can directly set the corresponding edges to zero. Denote *E*_*p*_ as the set of edges that are priorly known to be zero. Generally, we consider the following optimization problem.

(4) $$\begin{array}{c} \begin{array}{c} \min\\ \theta ,\eta \end{array}\left\{{-l}_{E}\left(\theta ,\eta \right)+\lambda_{n}\sum_{g=1}^{G}{\left\|\Theta_{g}\right\|}_{1,\text{off}}\right\}\\ \text{with}\;\Theta_{g}≻0,\Theta_{g,lm}=0\;\text{for}\;\left(l,m\right)\in E_{p},\eta \in H.\; \end{array}$$

We develop a block‐wise descent algorithm called VMPLN to optimize Eq. (4). The details of the optimization process of the VMPLN algorithm are shown in the Section 4.2. Generally speaking, VMPLN iteratively updates **P**, *π*, ${\left\{M_{g}\right\}}_{g=1}^{G}$, ${\left\{S_{g}\right\}}_{g=1}^{G}$ and ${\left\{\Theta_{g}\right\}}_{g=1}^{G}$ until the changes in the loss function and parameters between two successive update steps are small. Other than ${\left\{M_{g}\right\}}_{g=1}^{G}$, all other parameters can be optimized directly using either explicit updating formulas or available well‐performing optimization methods (Newton–Raphson algorithm and Glasso algorithm [9, 10]). The variational parameters ${\left\{M_{g}\right\}}_{g=1}^{G}$ are much more difficult to optimize (see formula (7) in the Section 4.2). Directly applying the p‐dimensional Newton–Raphson algorithm to optimize ${\left\{M_{g}\right\}}_{g=1}^{G}$ would involve calculating matrix inversions for nG different *p* × *p* matrices, making it computationally very expensive. To address this issue, we developed an efficient algorithm based on the alternating direction method of multipliers (ADMM) algorithm, which only needs to calculate matrix inversions for G different *p* × *p* matrices during optimization, making it computationally much more efficient.

We select the tuning parameter $\lambda_{n}>0$ for each $\Theta_{g}$ independently by minimizing the integrated complete likelihood criterion (ICL) [23]

(5) $$\begin{array}{c} -2l_{E}^{\left(g\right)}\left(\hat{\eta },\hat{\theta }\right)+\log\left({1_{n}}^{T}\;{\hat{P}}_{\cdot g}\right)\;s\left({\hat{\Theta }}_{g}\right), \end{array}$$

where $s\left({\hat{\Theta }}_{g}\right)$ denotes the number of nonzero elements in ${\hat{\Theta }}_{g}$. We can also select the tuning parameter *λ*_*n*_ such that the estimated network has the desired density.

### 2.2 VMPLN accurately recovered the network relationship for different populations in simulations

In this section, we perform simulation to evaluate the performance of VMPLN and compare with available graphical methods and single‐cell GRN estimation methods, including Glasso [10], LPGM [13], VPLN [15], PPCOR [24], GENIE3 [25] and PIDC [8]. For VMPLN, we use the clustering results given by the K‐means algorithm as the initial value and infer the networks jointly for all populations. For the other algorithms, we cluster the samples using the K‐means algorithm and infer a network for each cluster separately.

#### 2.2.1 Simulation setups

We first generate simulation data based on the MPLN model (See Section 2.1.1). The number of populations is set as G = 3 and the proportion parameter *π* is set as $\left(\frac{1}{3},\frac{1}{3},\frac{1}{3}\right)$. The number of observations is *n* = 3000. We consider 48 different simulation scenarios, which are 3 population‐mixing levels (low, middle, and high) × 2 dropout levels (i.e., the percent of zeros in data, low and high) × 2 dimension setups (*p* = 100, 300) × 4 graph structures. In each scenario, we generate 50 datasets, and the details of the data generation process are shown in the Supporting Information S1: Section S1.1. The four graph structures include:

1. Random graph: Pairs of nodes are connected with probability 0.1. Nonzero edges are randomly set as 0.3 or −0.3.
2. Hub graph: 20% of nodes are set as hub nodes. A hub node is connected to another node with probability 0.1. Non‐hub nodes are not connected to each other. Nonzero edges are randomly set as 0.3 or −0.3.
3. Blocked random graph: The nodes are divided into 5 blocks of equal size. Pairs of nodes within the same block are connected with probability 0.1. Nodes in different blocks are not connected. Nonzero edges are randomly set as 0.3 or −0.3.
4. Scale‐free graph: The Barabasi–Albert model [26] is used to generate a scale‐free graph with power 1. Nonzero edges are randomly set as 0.3 or −0.3.

To test VMPLN’s performance under the misspecified model setting, we also generate simulation data using a mixture multinomial log‐normal distribution. In this model, the conditional Poisson layer of the MPLN is replaced with a conditional multinomial distribution, which is as follows.

(6) $$\begin{array}{c} Y_{i}\;|\;X_{i}\;∼\;\text{Multinomial}\;\left(\lfloor \sum_{j=1}^{p}\lambda_{ij}\rfloor ,\frac{\lambda_{i}}{\sum_{j=1}^{p}\lambda_{ij}}\right), \end{array}$$

where *λ*_*ij*_ = *l*_*i*_exp(*X*_*i*,*j*_), *λ*_*i*_ = (*λ*_*i*1_,…,*λ*_*ip*_), and $\left\lfloor \sum_{j=1}^{p}\lambda_{ij}\right\rfloor$ represents the maximum integer that does not exceed $\sum_{j=1}^{p}\lambda_{ij}$. The simulation data are similarly generated.

#### 2.2.2 Performance comparison

The available methods often report network estimates with very different densities. Dense network predictions usually have a high sensitivity and a low specificity, but sparse network predictions have a low sensitivity and a high specificity. To make dense and sparse network predictions comparable, we adopt two criteria used in a previous benchmark work on network inference to evaluate the accuracy [27], called the pAUPRC ratio and the early precision ratio to evaluate the algorithms (See Section 4.3).

**FIGURE 2:**
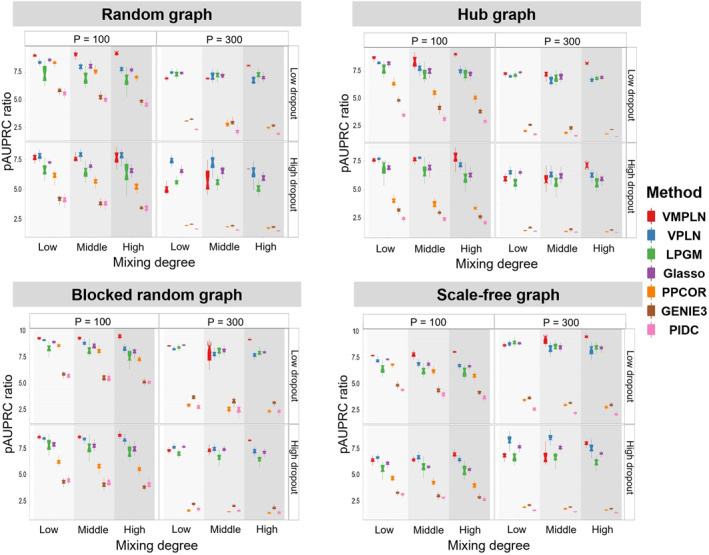
The pAUPRC ratios for four graphs. The parameters are set to their default values or tuned using their default methods.

We first compare the algorithms using their default parameters or default ways of selecting tuning parameters (see Supporting Information S1: Section S1.2 for details). Figure 2 shows the boxplots of pAUPRC ratios for different algorithms under different scenarios. Overall, VMPLN is the best‐performing algorithm in terms of the pAUPRC ratio. The advantage of VMPLN is more pronounced when the populations have a higher mixing level, suggesting that, compared with the two‐step procedure, the joint analysis of network inference and clustering can help to improve the network inference. The results for the early precision ratios are similar and shown in Figure S1a.

**FIGURE 3:**
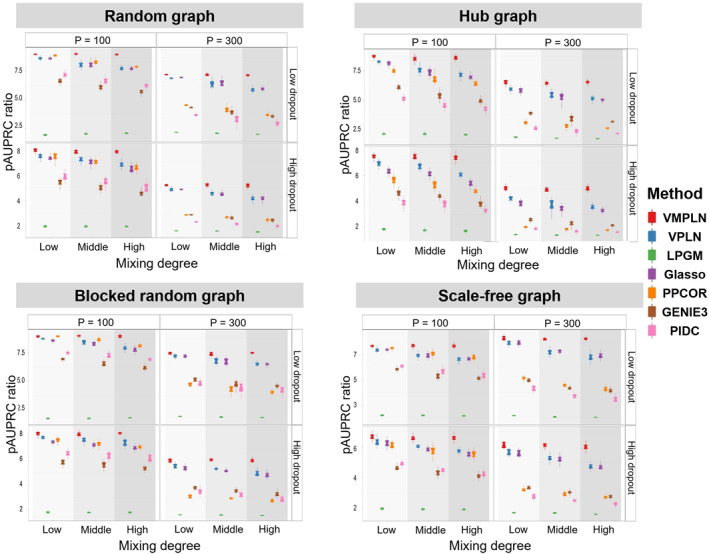
The pAUPRC ratios for four graphs. The edge score cutoffs or the tuning parameters are selected such that the network density is 20%.

Tuning parameter selection methods can have a large impact on the network estimates. To eliminate this influence, we tune the parameters such that the estimated networks from different algorithms have 20% nonzero edges (twice the density of the true network) (see Supporting Information S1: Section S1.2 for details), and compare their pAUPRC ratios (Figure 3) and early precision ratios (Figure S1b). Similarly, VMPLN has the best performance in most scenarios, especially in cases with high mixing levels. For example, in the simulation of the hub graph with *p* = 100 and low dropout, VMPLN has mean pAUPRC ratios of 8.63 and 8.72 in the high and low mixing scenarios, respectively, which are about 21% and 5% larger than the pAUPRC ratios (7.12 and 8.26) of VPLN under the same scenarios. All algorithms tend to have decreased performances in higher dimensions or with high dropout rates and VMPLN consistently has better performances in these more difficult settings.

The simulation results for the data generated from the misspecified model (i.e., the compositional model (6)) are shown in Figure S1C–F. Similarly, VMPLN also performs better in most simulation settings. We further assessed the memory and time efficiency across various methods. Tables S1 and S2 illustrate that VMPLN’s memory usage is moderate relative to competitors. Despite VMPLN incurring substantial time costs (Tables S3 and S4), its performance is justified. Unlike other methods that independently train the model parameters for pre‐clustering cell types and have fewer parameters, VMPLN iteratively updates parameters across all cell types. Notably, the efficient ADMM algorithm developed for the variational parameter ${\left\{M_{g}\right\}}_{g=1}^{G}$ optimization (see Section 4.2) enables VMPLN to maintain time costs comparable to VPLN, which has a much lower computational complexity. Additionally, VMPLN supports CPU parallel computation to boost training efficiency.

### 2.3 VMPLN accurately recovered the regulation relationship in scRNA‐seq benchmark data

In this section, we evaluate VMPLN and compare it with other state‐of‐the‐art GRN inference algorithms using two real scRNA‐seq datasets. One dataset is the scRNA‐seq of human peripheral blood mononuclear cells (PBMC) profiled by Ref. [28] (Kang dataset, 7217 cells from 10 cell types), and another dataset is the scRNA‐seq data of human PBMC cells profiled by Ref. [29] (Zheng dataset, 7242 cells from 6 cell types). The number of cells in each cell type in the two datasets is listed in Table S5. Figure S2 shows that there is a relatively high mixing degree between different cell types in these two benchmarking datasets. Both datasets consist of two batches. We use one of the two batches and the public GRN databases to construct silver standards (see Supporting Information S1: Sections S2.2–S2.3 for details), and test different algorithms using another batch. Regulatory relationships are inferred for the top 300, 400, and 500 highly variable genes selected by Seurat [30] and evaluated by comparing them with the silver standards. For algorithms other than VMPLN, the cell types are first identified using the K‐means algorithm, and gene regulatory relationships among highly variable genes are inferred for each cell type. We compare the algorithms at the same network density (5%) in terms of the pAUPRC ratio, the early precision ratio, and the stability. The pAUPRC ratios and early precision ratios are calculated by comparing them with the silver standard. The stability is defined as the median of pairwise Jaccard indexes between the networks estimated from 100 randomly down‐sampled (90%) datasets.

**FIGURE 4:**
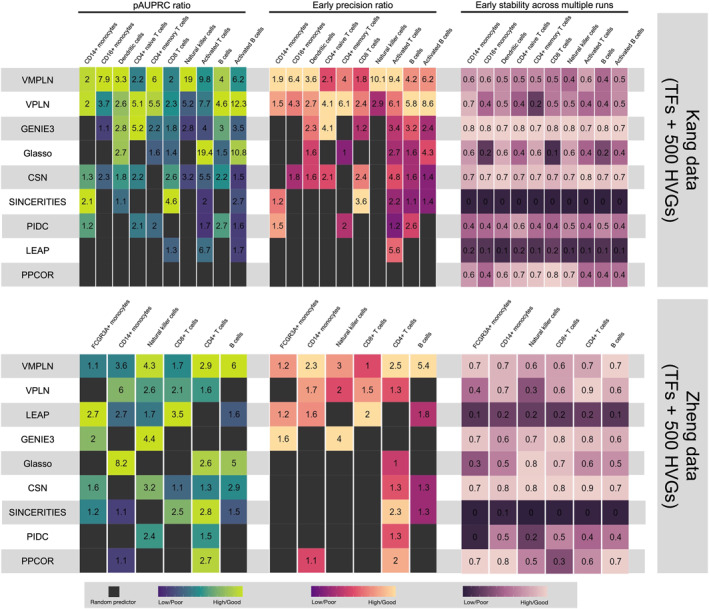
The performance of the network inference algorithms in two evaluation scRNA‐seq datasets using TFs and 500 most highly variable genes. The colors represent the scaled values of these metrics within each cell type, and the actual values are marked in the boxes. Black color in the boxes: the random predictor performs better. scRNA‐seq, single‐cell RNA sequencing.

Figure 4 shows the heatmap of the pAUPRC ratio, the early precision ratio, and the stability of the nine methods (seven methods evaluated in the simulation and two additional single‐cell GRN inference methods: CSN [31] and SINCERITIES [32]) for these two benchmarking datasets using TFs and 500 most highly variable genes. The algorithms are ordered by the average ranking of both pAUPRC ratios and early precision ratios across all cell types. In Kang dataset, VMPLN has the highest pAUPRC ratio, or highest early precision ratio, in 5/10 cell types, while the second‐best method, VPLN, has the highest pAUPRC ratio in 3/10 cell types and the highest early precision ratio in 4/10 cell types. In Zheng dataset, VMPLN has the highest pAUPRC ratio in 2/6 cell types and the highest early precision ratio in 3/6 cell types, while the second‐best method, VPLN, does not hold the highest ratio across all cell types. In addition, VMPLN is the only method that consistently outperforms the random predictor across all cell types. The stability of VMPLN is also reasonable and roughly similar to GENIE3. Note that GENIE3 is a tree‐ensemble‐based method that uses data perturbation for network estimation and thus should have good stability. Figure S3 showed the performance in two datasets using 300 and 400 most highly variable genes. VMPLN consistently outperforms other algorithms with higher average ranks of both pAUPRC ratios and early precision ratios across cell types, demonstrating robust stability.

### 2.4 VMPLN can be applied to identify the biological processes in COVID‐19 scRNA data

**FIGURE 5:**
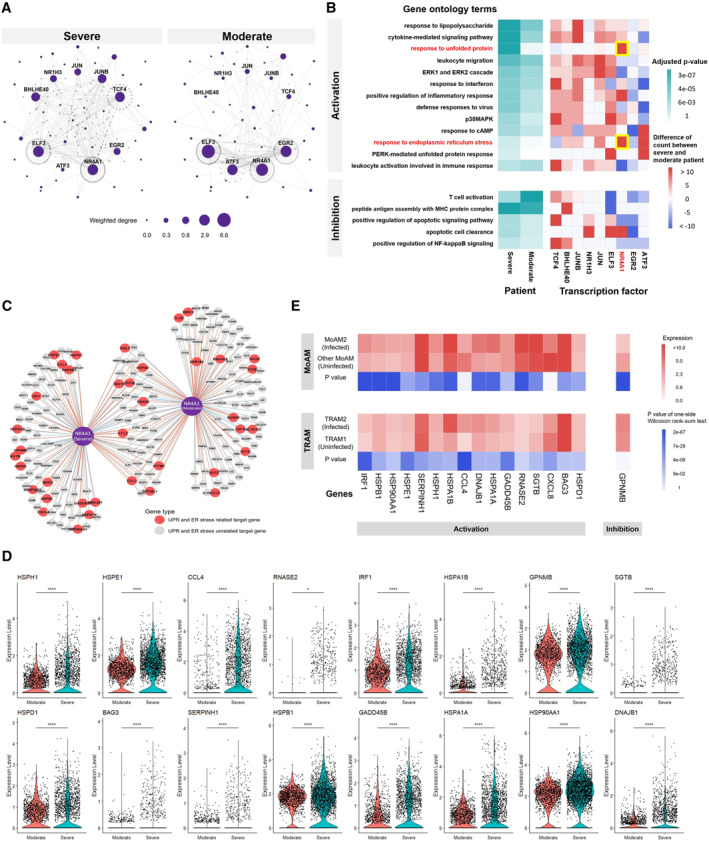
The GRN analysis of the COVID‐19 data. (A) The inferred gene regulatory networks of Group 4 macrophages in severe and moderate patients. The size of the node represents the weighted node degree. (B) Gene ontology enrichment analysis of transcription factors' target genes. Transcription factors are selected as those with a large weighted degree difference (>0.2) between severe and moderate patients. Activation and inhibition mean that the regulatory relationships are positive and negative, respectively. Left panel: *p*‐values of gene ontology terms. Right panel: differences in the number of genes in the gene ontology terms between severe and moderate patients. (C) The genes regulated by NR4A1. (D) The expression of the genes regulated by NR4A1 only in severe patients. “*”, *p* value < 0.05. “****”, *p* value < 10^−10^. (E) Differential gene expression analysis between cells with and without SARS‐CoV‐2 infection in macrophage single‐cell data from Ref. [34]. MoAM2, monocyte‐derived alveolar macrophage 2. TRAM, tissue‐resident alveolar macrophage 2. One‐sided Wilcoxon rank‐sum test is used. SARS‐CoV‐2, syndrome coronavirus 2. GRN, gene regulatory network.

We apply VMPLN to a SARS‐CoV‐2 dataset consisting of 29,980 bronchoalveolar lavage fluid macrophage cells from 8 patients, including 2 patients with moderate SARS‐CoV‐2 infection and 6 patients with severe infection. Liao et al. clustered the macrophages into four clusters [33], including two classic M1‐like macrophage groups (Group 1 and Group 2), the alternative M2‐like macrophages (Group 3), and the alveolar macrophages (Group 4). The four macrophages clusters seem to have a high mixing degree (Figure S4). The GRNs of the top 1000 highly variable genes for these four macrophage groups are inferred using VMPLN (Figure 5A and Figure S5). We focus on the alveolar macrophages (Group 4), since unlike other macrophage groups, the proportion of the alveolar macrophages (Group 4) tends to be smaller in patients with severe infection [33].

Various transcription factors exhibit a large weighted degree (weighted by absolute partial correlations) difference between the GRNs in the moderate and severe patients (Figure S6). Gene ontology enrichment analysis of their target genes (Figure 5B) shows that, as expected, many target genes are involved in immune responses such as leukocyte migration and regulation of T cell activation. Interestingly, we observe that several gene ontology terms are only enriched in severe patients, such as response to unfolded protein (UPR) and response to endoplasmic reticulum (ER) stress, mainly due to the activation of target genes of NR4A1 (Figure 5B). Moreover, the expression of genes regulated by NR4A1 only in severe patients are significantly upregulated in severe patients compared with moderate patients (Figure 5D). Similar analyses of the other macrophage groups also show that UPR is more enriched in severe patients than in moderate patients (Figure S7).

The UPR and ER stress processes are frequently activated in cells infected by viruses [35] including coronavirus [36], indicating that alveolar macrophages might be infected by SARS‐CoV‐2. In fact, a recent study showed that macrophages can be infected by SARS‐CoV‐2, and the infected macrophages activate T cells to promote alveolitis in patients with severe COVID‐19 [34]. These studies imply that the UPR and ER stress‐related genes might be activated in SARS‐CoV‐2‐infected macrophages through modulation of TFs such as NR4A1. Macrophage single‐cell data in Ref. [34] has SARS‐CoV‐2 infection information for each single cell and thus allows expression comparison between cells with or without SARS‐CoV‐2 infection. We take the UPR and ER stress‐related genes that are regulated by NR4A1 only in severe patients (Figure 5C) and compare their expressions between cells with and without SARS‐CoV‐2 infection in macrophage single‐cell data from Ref. [34]. We find that most of these genes are indeed significantly differentially expressed (Figure 5E). In light of these findings, we reason that NR4A1 might play an important role in regulating cellular responses to the SARS‐CoV‐2 infection. A number of NR4A1’s target genes, including IRF1 and HSP90, have recently been discovered to be potential therapeutic targets for COVID‐19 [37, 38]. NR4A1 and its target genes that we identified here may also serve as potential therapeutic targets.

## 3 DISCUSSION

In this paper, we develop a regulatory network inference method called VMPLN for scRNA‐seq data. Instead of using the two‐step procedure for network inference, VMPLN performs clustering and network inference simultaneously and is thus especially suitable for scRNA‐seq with mixed cell types. Most of the scRNA‐seq data contains multiple cell types. We expect that VMPLN will have many applications in single‐cell studies.

A potential limitation of VMPLN is that it assumes that the regulatory relationships are linear. If the regulatory relationships are far from being linear, VMPLN will not perform well. Methods such as GENIE3 can allow the regulatory relationships to be nonlinear, but they require pre‐clustering before network inference. One important research direction is to develop network methods that can account for nonlinear regulatory relationships as well as mixed cell populations. In addition, VMPLN is developed for scRNA‐seq data. Other data and information can only be incorporated by setting the prior edges. Currently, single‐cell multi‐omics technologies have been developed [39]. Developing network inference methods that can integrate multi‐omics data can help to improve sensitivity and reduce false discoveries. Another limitation of VMPLN is determining the optimal number of cell types, which presents a significant challenge in analyzing scRNA‐seq data [40]. We could use pre‐clustering and biological priors to guide the selection of cell type numbers, or alternatively, we could further enhance the mixture‐PLN model by incorporating Dirichlet process mixtures [41] or other criteria that enable the joint inference of the cell type number.

## 4 MATERIALS AND METHODS

### 4.1 Datasets

To train and evaluate our model, we collected three benchmark single‐cell RNA‐seq datasets, including Kang dataset [28], Zheng dataset [29], and Liao dataset [33]. The Kang dataset was derived from Ref. [28], which is the scRNA‐seq data of human PBMC, and contains 7217 cells from 10 cell types. The Zheng dataset was derived from Ref. [29], which is the scRNA‐seq data of PBMC and contains 7424 cells from 6 cell types. The Liao dataset was derived from Ref. [33], which is the scRNA‐seq data of 29,980 bronchoalveolar lavage fluid macrophage cells from eight patients, including two patients with moderate SARS‐CoV‐2 infection and six patients with severe infection. More details about the collected datasets can be found in Supporting Information S1. To support the identified biological processes in the SARS‐CoV‐2 study, we also collect a single‐cell RNA‐seq dataset, which has SARS‐CoV‐2 infection information for each single cell [34]. All data used are available and can be accessed at zenodo website (records/7069698).

### 4.2 The optimization process of the VMPLN algorithm

We develop a block‐wise descent algorithm called VMPLN to optimize (4). Let

$$L_{1}\left(M_{g,i\cdot },\Theta_{g},\mu_{g}\right)=\frac{{\left(M_{g,i\cdot }-\;\mu_{g}\right)}^{T}\Theta_{g}\left(M_{g,i\cdot }-\;\mu_{g}\right)}{2},$$

$$L_{2}\left(M_{g,ij},l_{i},S_{g,ij}\right)=-\Lambda_{g,ij}^{\left(1\right)}+F_{1}\left(l_{i},M_{g,ij},S_{g,ij}^{\left(k+1\right)}\right),$$

where $\Lambda_{g,ij}^{\left(1\right)}=Y_{ij}\times M_{g,ij,}F_{1}\left(l_{i},M_{g,ij},S_{g,ij}^{\left(k+1\right)}\right)=\exp\left(M_{g,ij}+\frac{1}{2}S_{g,ij}+\log\;l_{i}\right).$

Given the initial values, we iteratively update **P**, *π*, ${\left\{M_{g}\right\}}_{g=1}^{G}$, ${\left\{S_{g}\right\}}_{g=1}^{G}$, μ, and ${\left\{\Theta_{g}\right\}}_{g=1}^{G}$ and terminate the iteration if changes between two successive update steps are small. The VMPLN algorithm is summarized in Algorithm 1. The parameters **P**, *π*, and μ all have explicit updating formulas and can be efficiently calculated. For the parameter **S**, given all other parameters, the loss function can be decomposed into a sum of npG functions, each of which only involves one *S*_*g*,*ij*_ and can be efficiently solved by the Newton–Raphson algorithm. For the network parameters Θ_*g*_ (*g* = 1,…,*G*), given all other parameters, the corresponding sub‐optimization problem is equivalent to solving G independent Glasso problems [9, 10].

For the parameters M, the sub‐optimization problem corresponding to $M_{g}\;\left(g=1,\text{…},G\right)$ is

(7) $$\begin{array}{c} \begin{array}{c} \mathrm{argmin}\\ {\left\{M_{g}\right\}}_{g=1}^{G} \end{array}\left\{\sum_{g=1}^{G}\sum_{i=1}^{n}P_{ig}\left\{L_{1}\left(M_{g,i\cdot },{\hat{\Theta }}_{g}^{\left(k\right)},{\hat{\mu }}_{g}^{\left(k\right)}\right)+\sum_{j=1}^{p}L_{2}\left(M_{g,ij},l_{i},{\hat{S}}_{g,ij}^{\left(k\right)}\right)\right\}\right\}, \end{array}$$

which is equivalent to nG independent optimization problems (*) in Algorithm 1. We develop an efficient algorithm based on the ADMM algorithm [42] to optimize (*) in Algorithm 1. Specifically, we introduce an auxiliary matrix **N**_*g*_ for **M**_*g*_, and denote:

$$L_{M}\left(M_{g,i\cdot },N_{g,i\cdot }\right)=L_{1}\left(N_{g,i\cdot },{\hat{\Theta }}_{g}^{\left(k\right)},{\hat{\mu }}_{g}^{\left(k\right)}\right)+\sum_{j=1}^{p}L_{2}\left(M_{g,ij},l_{i},{\hat{S}}_{g,ij}^{\left(k\right)}\right).$$

Solving (*) in Algorithm 1 is equivalent to solving the following problem

(8) $$\begin{array}{c} \begin{array}{c} \mathrm{argmin}\\ M_{g,i\cdot },N_{g,i\cdot } \end{array}\left\{L_{M}\left(M_{g,i\cdot },N_{g,i\cdot }\right)\right\}\;\text{with}\;M_{g,i\cdot }=N_{g,i\cdot }\;. \end{array}$$

The augmented Lagrangian of the optimization problem (8) is as follows

$$L_{M}\left(M_{g,i\cdot },N_{g,i\cdot }\right)+\sum_{j=1}^{p}\alpha_{j}\left(M_{g,ij}-N_{g,ij}\right)+\frac{\rho }{2}\sum_{j=1}^{p}\alpha_{j}{\left(M_{g,ij}-N_{g,ij}\right)}^{2},$$

where *α* = (*α*_1_,…,*α*_*p*_) is the Lagrangian multiplier, and *ρ* is the step size. Given initial values, we iteratively update **M**_*g*,*i*⋅_, **N**_*g*,*i*⋅,_ and *α* (Algorithm 2). The optimization problem for **M**_*g*,*i*⋅_ can be decomposed into *p* independent one‐dimensional optimization problems, which can be easily optimized by the one‐dimensional Newton–Raphson algorithm (Step 1 in Algorithm 2). The optimization problem for **N**_*g*,*i*⋅_ has an explicit solution and involves inverting the matrix $\rho I+{\hat{\Theta }}_{g}^{\left(k\right)}$ (Step 2 in Algorithm 2). We only need to invert the matrix $\rho I+{\hat{\Theta }}_{g}^{\left(k\right)}$ once. In total, the **M**‐step of Algorithm 1 involves nG optimization problems (*) in Algorithm 1, but we only need to calculate *p* × *p* matrix inversions G times in the nG runs of Algorithm 2.

To initialize the parameters in Algorithm 1, we perform dimension reduction using principal component analysis on the normalized data $\tilde{Y}=\log\frac{Y+1}{\hat{l}{\;1}_{p}^{T}}$ with ${\hat{l}}_{i}=\sum_{j=1}^{p}\frac{Y_{ij}}{10^{4}}\;\left(i=1,2,\text{…},n\right)$. Then, we use K‐means [43] to cluster single cells in the low‐dimensional space. We conduct three random starts to optimize clustering assignment and ensure convergence. Let ${\tilde{Z}}_{i}\in \left\{1,2,\text{…},G\right\}$ be the clustering label of the *i*th cell. The parameters are initialized as:

$${\hat{P}}_{ig}^{\left(0\right)}=I\left({\tilde{Z}}_{i}=g\right),{\hat{M}}_{g,ij}^{\left(0\right)}={\tilde{Y}}_{ij},{\hat{S}}_{g,ij}^{\left(0\right)}=10^{-5},$$

$${\hat{\pi }}_{g}^{\left(0\right)}=\frac{1}{n}\sum_{i=1}^{n}{\hat{P}}_{ig}^{\left(0\right)},{\hat{\mu }}_{gj}^{\left(0\right)}={\left(\sum_{i=1}^{n}{\hat{P}}_{ig}^{\left(0\right)}\right)}^{-1}\sum_{i=1}^{n}{\hat{P}}_{ig}^{\left(0\right)}{\hat{M}}_{g,ij}^{\left(0\right)},$$

withg=1,…,G;i=1,…,n;j=1,…,p.

The precision matrix ${\hat{\Theta }}_{g}^{\left(0\right)}$ of the *g*th cell type is initialized by the Glasso algorithm with its penalty parameter as 10^−6^ and its covariance matrix ${\hat{\Sigma }}_{g}^{\left(0\right)}$, which is as follows.

$${\hat{\Sigma }}_{g}^{\left(0\right)}=\frac{\sum_{i=1}^{n}{\hat{P}}_{ig}^{\left(0\right)}\left\{{\hat{M}}_{g,i\cdot }^{\left(0\right)}{{\hat{M}}_{g,i\cdot }^{\left(0\right)}}^{T}+D\left({\hat{S}}_{g,i\cdot }^{\left(0\right)}\right)\right\}}{\sum_{i=1}^{n}{\hat{P}}_{ig}^{\left(0\right)}}.$$

Algorithm 1. **Framework of VMPLN.**

1

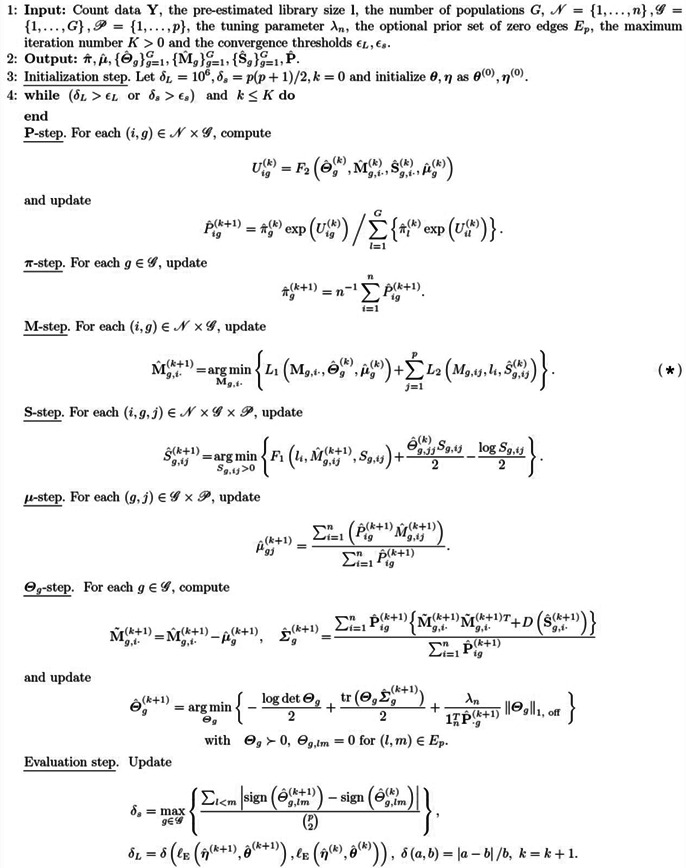

Algorithm 2. **ADMM algorithm for updating $M_{g,i}$.**

1

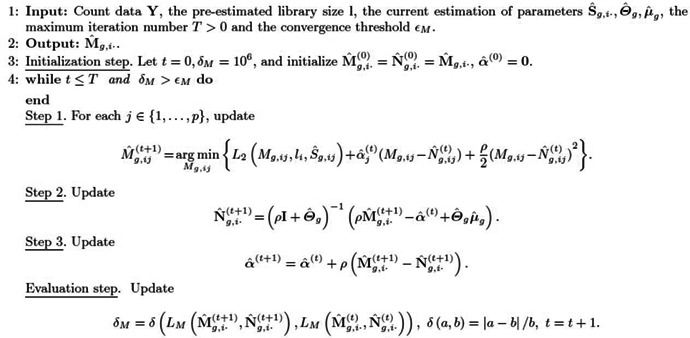

### 4.3 The pAUPRC ratio and early precision ratio

Briefly, given a network estimation by an algorithm, we calculate its area under the partial precision‐recall curve (called pAUPRC) by applying varying thresholds to the edge scores given by the algorithm (see Supporting Information S1: Section S1.2 for details). The pAUPRC ratio is defined as the ratio between the pAUPRC and the expected pAUPRC of the random network prediction of the same density. The early precision is defined as the precision of the top min{K,s} edges in the inferred network, where K is the number of edges in the true network and s is the number of edges in the inferred network. The early precision ratio is defined as the ratio between the early precision of the algorithm and the early precision of the random network prediction at the same density.

## AUTHOR CONTRIBUTIONS

**Junjie Tang**: Conceptualization; formal analysis; methodology; software; validation; visualization; writing – original draft. **Changhu Wang**: Formal analysis; methodology; software. **Feiyi Xiao**: Conceptualization; methodology. **Ruibin Xi**: Conceptualization; funding acquisition; methodology; project administration; supervision; writing – original draft; writing – review & editing.

## CONFLICT OF INTEREST STATEMENT

The authors Junjie Tang, Changhu Wang, Feiyi Xiao, and Ruibin Xi declare that they have no conflicts of interest.

## ETHICS STATEMENT

This article does not contain any studies with human or animal subjects performed by any of the authors.

## Supporting information

Supporting Information S1

## Data Availability

VMPLN is implemented in R (github.com/XiDsLab/SCVMPLN). All data used and corresponding source codes are available and can be accessed at zenodo website (records/7069698).
