# Supplementary material for: Single‐cell gene regulatory network analysis for mixed cell populations
Source: Quant Biol. 2024 Jul 2;12(4):375–88. doi: 10.1002/qub2.64 (PMC12806009; doi:10.1002/qub2.64)
Supplement: Supplementary file 1 — Supporting Information S1 [file QUB2-12-375-s001.docx]

Supplementary Material for “VMPLN: Single-cell gene regulatory network analysis for mixed cell populations”

Junjie Tang^1,†^, Changhu Wang^1,†^, Feiyi Xiao^1^, and Ruibin Xi^1,2,*^

^1^ School of Mathematical Sciences and Center of Statistical Science, Peking University, Beijing 100871, China

^2^ Academy for Advanced Interdisciplinary Studies, Peking University, Beijing 100871, China

† Equal contribution.

^*^ Corresponding author. E-mail: ruibinxi@math.pku.edu.cn

**S1 Supplementary: Simulation**

**S1.1 Details of the Data Generation Process**

For each simulation dataset, we first independently generate the precision matrix for each of the three latent normal distributions according to one of the four graph structures. When generating the precision matrices, the diagonal elements are set to 1 plus a small positive number to guarantee positive definiteness. Then, we generate the mean vectors $\boldsymbol{\mu}_{1},\boldsymbol{\mu}_{2},\boldsymbol{\mu}_{3}$ for the latent normal distributions. The first $p_{d}$ elements of $\boldsymbol{\mu}_{g} (g=1,2,3)$ are independently sampled from $\left\{ v_{1},\frac{v_{1}+v_{2}}{2},v_{2} \right\}$. The remaining ${p-p}_{d}$ elements are shared among $\boldsymbol{\mu}_{1},\boldsymbol{\mu}_{2},\boldsymbol{\mu}_{3}$ and are independently sampled from $\left\{ v_{3},v_{4} \right\}$. We set $\left( {v_{1},v_{2},v}_{3},v_{4} \right)$ as  $\left( 2.4,-0.1,0.9,-0.1 \right)$ in the low dropout case (about 10% zeros) and $\left( 1.4,-1.1,-0.1,-1.1 \right)$ in the high dropout case (about 40% zeros). We vary $p_{d}$ to control the mixing degree of the three populations. The scaling factors $\mathbf{l}\boldsymbol{=}\left( l_{1},\ldots,l_{n} \right)$are independently generated from a log-normal distribution $\log N\left( \log10,0.05 \right)$. With these model parameters, we finally generate the observed expression $\mathbf{Y}_{1},\ldots,\mathbf{Y}_{n}$ from the mixture Poisson log-normal model. We calculate the Adjusted Rand Index between the true population label and the population label from the K-means clustering ([1](#_ENREF_1)) of the normalized data $\tilde{\mathbf{Y}}=\log\frac{\mathbf{Y+1}}{\hat{\mathbf{l}}\boldsymbol{1}_{p}^{T}}$ with $\hat{l}_{i}= \sum_{j=1}^{p} \frac{Y_{ij}}{{10}^{4}} (i=1,2,\ldots,n)$. We vary $p_{d}$ such that the low-level mixing data have an adjusted rand index value in  $(0.9,1]$, the middle-level mixing data have an Adjusted Rand Index value in $(0.75,0.85]$ and the high-level mixing data have an adjusted rand index value in $(0.65,0.75]$.

**S1.2 The Description of Edge Scores, Partial Precision-Recall Curve, and Parameter Selection for Different Algorithms**

1. **Edge scores:**

We define the edge scores to represent the connective strength of edges for different algorithms. For VMPLN, VPLN, and Glasso, suppose that $\hat{\boldsymbol{\Theta}}$ is its estimation of a network, and we define an edge score for the edge $\left( i,j \right)(i\neq j)$ as its absolute partial correlation (i.e., $\left| -\left( \hat{\Theta}_{ii} \hat{\Theta}_{jj} \right)^{-\frac{1}{2}}\hat{\Theta}_{ij} \right|$). For LPGM, we define an edge score for each edge as its stability score. For PPCOR, GENIE3, and PIDC, we define an edge score for each edge as its estimated connected weight.

1. **Partial precision-recall curve:**

Since the network inferred by the available method contains connected edges with different edge scores and unconnected edges with zero edge scores, the precision-recall curve constructed by varying the threshold of the edge scores is incomplete, and we can only obtain a partial precision-recall curve. We calculate its area under this partial precision-recall curve (pAUPRC). In order to eliminate the influence of different network densities given by the available methods, we further define the pAUPRC ratio as the ratio between the pAUPRC and the expected pAUPRC of the random network prediction with the same network density for a fair comparison.

1. **Parameter selection for different algorithms:**
   1. **Selecting parameters using each algorithm's default**

We use default parameters for PPCOR, GENIE3, and PIDC. We tune the parameters of VMPLN using the integrated complete likelihood criterion, the parameters of VPLN and Glasso using the Bayesian information criterion, and the parameters of LPGM using the stability criterion.

- 1. **Selecting parameters such that the network density is 20%**

VMPLN, VPLN, Glasso, and LPGM, we first tune their tuning parameters such that the densities of the estimated networks are 20%. For PPCOR, GENIE3, and PIDC, we select the edge score cutoffs such that the estimated network densities are 20%.

**S1.3 Additional Result of Simulation**

| **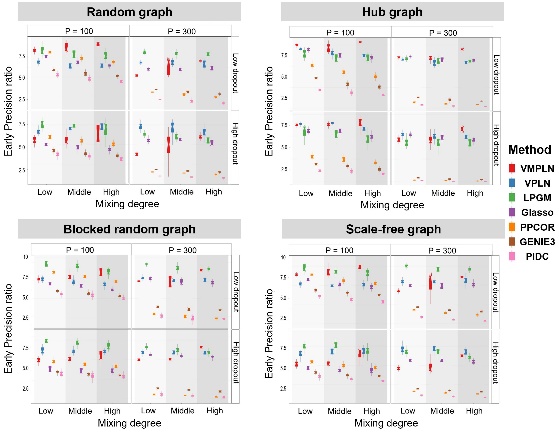**   1. The early precision ratios for four graphs. The parameters are set to their default values or tuned using their default methods. | **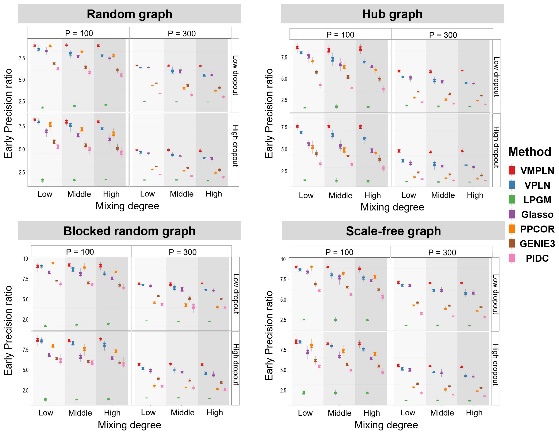**   1. The early precision ratios for four graphs. The edge score cutoffs or the tuning parameters are selected such that the network density is 20%. |
| --- | --- |
| **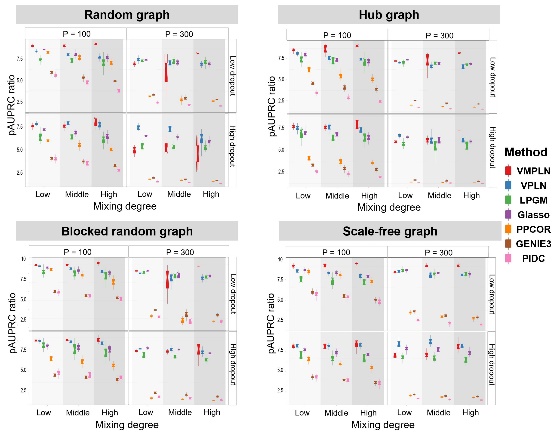**   1. The pAUPRC ratios for four graphs and the data generated from the compositional model. The parameters are set to their default values or tuned using their default methods. | **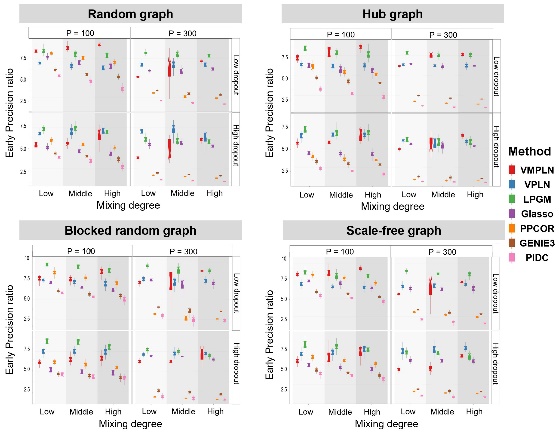**   1. The early precision ratios for four graphs and the data generated from the compositional model. The parameters are set to their default values or tuned using their default methods. |
| **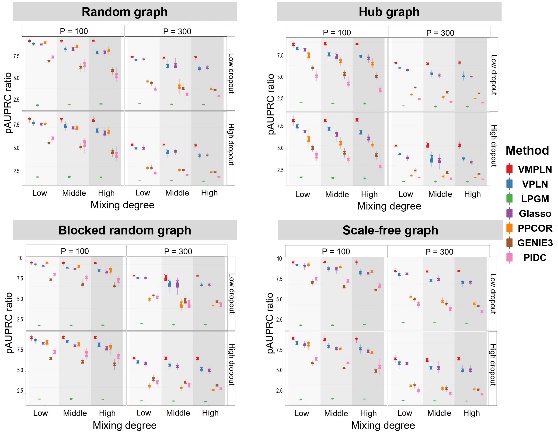**   1. The pAUPRC ratios for four graphs and the data generated from the compositional model. The edge score cutoffs or the tuning parameters are selected such that the network density is 20%. | **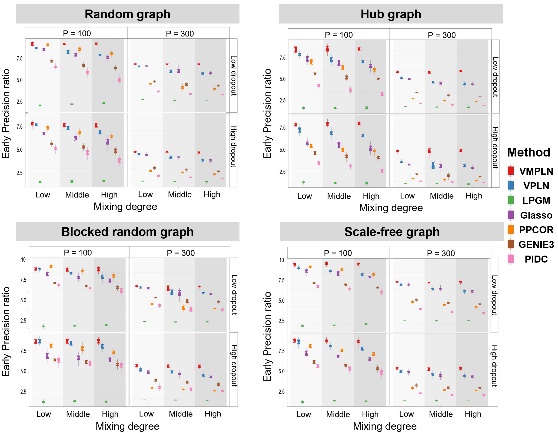**   1. The early precision ratios for four graphs and the data generated from the compositional model. The edge score cutoffs or the tuning parameters are selected such that the network density is 20%. |

**Fig. S1 Additional Result of pAUPRC Ratios and Early Precision Ratios in Simulation.**

Table. S1, Table. S2, Table. S3 and Table. S4 show the computational memory usage and time cost of the seven algorithms under the computer configuration of Linux OS, Intel(R) Xeon(R) Gold 6132 CPU @ 2.60GHz and 10G RAM.

| Dimension | p=100 | | | p=100 | | |
| --- | --- | --- | --- | --- | --- | --- |
| Dropout | Low | | | High | | |
|  | Random graph | | | | | |
| Mixing degree | Low | Middle | High | Low | Middle | High |
| VMPLN | 153.9 (18.5) | 167.1 (23.2) | 171.7 (22.8) | 154.6 (18.6) | 163.5 (22.8) | 167.6 (23.2) |
| VPLN | 660.7 (14.3) | 664.2 (27.6) | 747.4 (58.2) | 664.2 (12.3) | 649.2 (24.6) | 702.5 (48.5) |
| LPGM | 136.3 (23.8) | 149.9 (21.2) | 119.2 (3.2) | 118.8 (3.4) | 126.9 (3.3) | 130.4 (3.3) |
| Glasso | 186.6 (0.2) | 186.6 (0.2) | 186.6 (0.3) | 186.7 (0.2) | 186.7 (0.2) | 186.6 (0.2) |
| PPCOR | **8.1 (0.0)** | **8.1 (0.1)** | **8.1 (0.0)** | **8.1 (0.0)** | **8.1 (0.0)** | **8.1 (0.0)** |
| GENIE3 | 42.5 (0.5) | 42.4 (0.5) | 42.5 (0.8) | 42.5 (0.5) | 42.6 (0.5) | 42.5 (0.7) |
| PIDC | 885.4 (82.3) | 853.8 (5.9) | 855.1 (7.2) | 819.1 (2.6) | 818.8 (2.2) | 818.2 (2.8) |
|  | Hub graph | | | | | |
| VMPLN | 153.9 (18.6) | 171.7 (22.7) | 176.2 (21.2) | 167.8 (23.2) | 158.9 (21.3) | 166.9 (0.1) |
| VPLN | 666.6 (9.3) | 691.4 (14.8) | 768.8 (49.8) | 658.4 (15.1) | 664.1 (18.9) | 713.5 (65.7) |
| LPGM | 118.8 (3.3) | 159.1 (11.2) | 164.1 (3.5) | 121.4 (3.2) | 125.7 (3.2) | 123.3 (3.3) |
| Glasso | 186.6 (0.3) | 186.7 (0.2) | 186.7 (0.4) | 186.7 (0.2) | 186.7 (0.3) | 186.5 (0.4) |
| PPCOR | **8.1 (0.1)** | **8.1 (0.0)** | **8.1 (0.0)** | **8.1 (0.0)** | **8.1 (0.0)** | **8.1 (0.0)** |
| GENIE3 | 42.6 (0.5) | 42.7 (0.6) | 42.5 (0.9) | 42.6 (0.4) | 42.5 (0.5) | 42.2 (0.5) |
| PIDC | 827.2 (5.3) | 824.1 (3.5) | 822.4 (5.6) | 803.8 (3.2) | 803.4 (2.8) | 801.4 (2.2) |
|  | Blocked random graph | | | | | |
| VMPLN | 149.5 (13.9) | 162.7 (22.7) | 171.6 (22.8) | 154.5 (18.6) | 150.3 (14.0) | 172.4 (22.7) |
| VPLN | 663.6 (12.4) | 687.1 (55.4) | 725.9 (53.2) | 664.1 (12.5) | 664.3 (26.7) | 691.8 (54.8) |
| LPGM | 118.8 (3.3) | 119.8 (3.4) | 120.9 (3.3) | 124.5 (3.3) | 123.0 (3.2) | 125.2 (3.3) |
| Glasso | 186.6 (0.2) | 186.7 (0.2) | 184.1 (7.9) | 186.8 (0.2) | 184.2 (7.7) | 184.3 (7.7) |
| PPCOR | **8.1 (0.0)** | **8.1 (0.0)** | **8.1 (0.0)** | **8.1 (0.0)** | **8.1 (0.1)** | **8.1 (0.0)** |
| GENIE3 | 42.5 (0.5) | 42.4 (0.5) | 42.4 (0.7) | 42.5 (0.5) | 42.6 (0.5) | 42.4 (0.5) |
| PIDC | 860.2 (4.1) | 857.2 (6.5) | 859.5 (8.1) | 822.0 (3.0) | 822.3 (2.8) | 821.8 (4.9) |
|  | Scale-free graph | | | | | |
| VMPLN | 149.6 (13.9) | 158.5 (21.2) | 184.7 (13.9) | 145.8 (0.1) | 159.1 (21.3) | 172.3 (22.8) |
| VPLN | 654.9 (15.5) | 696.0 (45.9) | 783.7 (57.6) | 652.5 (20.4) | 664.2 (12.5) | 748.5 (65.4) |
| LPGM | 118.8 (3.3) | 124.5 (3.4) | 124.2 (3.2) | 124.3 (3.3) | 126.3 (3.3) | 131.5 (3.2) |
| Glasso | 186.7 (0.3) | 186.7 (0.2) | 186.8 (0.3) | 186.7 (0.2) | 186.7 (0.2) | 186.8 (0.3) |
| PPCOR | **8.1 (0.0)** | **8.1 (0.0)** | **8.1 (0.0)** | **8.1 (0.0)** | **8.1 (0.0)** | **8.1 (0.0)** |
| GENIE3 | 42.5 (0.4) | 42.4 (0.5) | 42.3 (0.6) | 42.5 (0.4) | 42.5 (0.3) | 42.5 (0.8) |
| PIDC | 852.3 (6.3) | 848.2 (5.5) | 844.7 (7.0) | 816.6 (3.9) | 814.7 (4.2) | 813.0 (1.2) |

**Table. S1: Comparison of GRN inference methods in terms of memory usage (MiB) for p = 100 in simulation.** The results are averages over 50 replicates with standard deviations in brackets.

| Dimension | p=300 | | | p=300 | | |
| --- | --- | --- | --- | --- | --- | --- |
| Dropout | Low | | | High | | |
|  | Random graph | | | | | |
| Mixing degree | Low | Middle | High | Low | Middle | High |
| VMPLN | 607.8 (92.7) | 649.2 (97.4) | 706.2 (1.2) | 564.6 (103.7) | 585.1 (79.3) | 686.2 (51.3) |
| VPLN | 2133.7 (34.8) | 2315.9 (169.1) | 2514.2 (104.7) | 2215.5 (109.9) | 2169.9 (130.8) | 2387.4 (186.0) |
| LPGM | 742.8 (40.7) | 691.4 (31.6) | 744.0 (48.8) | 737.3 (51.2) | 668.5 (32.2) | 655.2 (32.1) |
| Glasso | 491.9 (9.5) | 493.9 (34.3) | 498.4 (20.8) | 483.1 (52.1) | 457.4 (21.8) | 484.4 (26.7) |
| PPCOR | **56.7 (0.0)** | **56.8 (0.0)** | **56.8 (0.1)** | **56.7 (0.1)** | **56.8 (0.1)** | **56.7 (0.1)** |
| GENIE3 | 114.8 (2.8) | 115.3 (2.7) | 114.7 (2.0) | 112.3 (5.0) | 112.8 (4.8) | 113.9 (2.8) |
| PIDC | 19054.5 (22.3) | 19060.9 (43.6) | 19004.4 (39.4) | 19074.7 (17.8) | 19099.2 (31.9) | 19075.2 (24.7) |
|  | Hub graph | | | | | |
| VMPLN | 596.2 (56.9) | 661.6 (67.5) | 705.5 (0.6) | 593.4 (98.1) | 578.4 (91.8) | 664.9 (68.2) |
| VPLN | 2094.2 (39.0) | 2285.8 (164.8) | 2563.7 (111.7) | 2220.5 (116.8) | 2240.0 (129.3) | 2453.6 (208.4) |
| LPGM | 651.2 (32.6) | 701.2 (31.8) | 654.2 (31.1) | 724.7 (54.9) | 693.4 (69.5) | 744.1 (52.9) |
| Glasso | 496.6 (1.6) | 481.4 (27.1) | 503.1 (29.1) | 457.7 (29.2) | 478.2 (41.9) | 485.4 (39.3) |
| PPCOR | **56.8 (0.1)** | **56.7 (0.1)** | **56.7 (0.0)** | **56.8 (0.0)** | **56.8 (0.0)** | **56.7 (0.1)** |
| GENIE3 | 114.6 (2.8) | 115.4 (2.5) | 115.2 (1.6) | 115.1 (2.6) | 113.9 (1.2) | 114.1 (2.4) |
| PIDC | 18828.7 (27.9) | 18812.0 (26.9) | 18752.9 (39.3) | 18854.0 (41.7) | 18844.8 (24.1) | 18835.3 (35.0) |
|  | Blocked random graph | | | | | |
| VMPLN | 618.8 (73.8) | 573.0 (102.0) | 689.3 (49.9) | 636.1 (83.3) | 591.9 (99.5) | 685.2 (50.9) |
| VPLN | 2126.8 (43.3) | 2353.8 (240.8) | 2629.8 (177.2) | 2122.0 (86.6) | 2220.5 (153.5) | 2503.6 (255.1) |
| LPGM | 741.8 (42.8) | 742.1 (45.4) | 722.5 (48.8) | 733.9 (48.4) | 739.3 (55.7) | 739.1 (46.6) |
| Glasso | 491.6 (8.9) | 465.9 (23.9) | 478.0 (23.0) | 497.1 (44.6) | 464.3 (21.3) | 491.1 (37.7) |
| PPCOR | **56.8 (0.1)** | **56.8 (0.1)** | **56.7 (0.1)** | **56.7 (0.1)** | **56.8 (0.0)** | **56.8 (0.1)** |
| GENIE3 | 115.1 (2.5) | 115.3 (2.8) | 115.6 (3.6) | 114.8 (2.6) | 114.9 (2.3) | 115.8 (3.3) |
| PIDC | 19088.6 (33.6) | 19075.3 (28.4) | 19006.8 (61.1) | 19102.7 (33.3) | 19103.8 (36.5) | 19069.2 (44.1) |
|  | Scale-free graph | | | | | |
| VMPLN | 580.3 (100.0) | 643.9 (78.5) | 705.4 (0.8) | 635.9 (84.0) | 603.2 (84.1) | 664.7 (70.0) |
| VPLN | 2168.3 (68.2) | 2335.4 (148.3) | 2491.6 (262.6) | 2155.4 (130.5) | 2270.5 (135.5) | 2408.5 (209.2) |
| LPGM | 738.2 (23.8) | 701.3 (31.2) | 751.7 (56.4) | 735.6 (44.4) | 732.2 (57.2) | 695.4 (30.9) |
| Glasso | 494.4 (33.0) | 474.8 (25.8) | 482.9 (14.6) | 483.2 (37.6) | 466.9 (44.3) | 483.1 (30.9) |
| PPCOR | **56.8 (0.1)** | **56.7 (0.0)** | **56.8 (0.0)** | **56.8 (0.1)** | **56.8 (0.0)** | **56.8 (0.1)** |
| GENIE3 | 114.8 (1.9) | 116.1 (5.0) | 116.3 (4.3) | 114.9 (1.9) | 115.7 (4.5) | 115.0 (2.5) |
| PIDC | 19061.4 (38.4) | 19027.1 (52.8) | 19005.8 (33.7) | 19090.0 (35.5) | 19105.4 (38.2) | 19060.6 (34.3) |

**Table. S2: Comparison of GRN inference methods in terms of memory usage (MiB) for p = 300 in simulation.** The results are averages over 50 replicates with standard deviations in brackets.

| Dimension | p=100 | | | p=100 | | |
| --- | --- | --- | --- | --- | --- | --- |
| Dropout | Low | | | High | | |
|  | Random graph | | | | | |
| Mixing degree | Low | Middle | High | Low | Middle | High |
| VMPLN | 70.0 (5.3) | 77.1 (18.3) | 93.0 (14.7) | 81.1 (4.9) | 86.7 (6.6) | 105.7 (7.7) |
| VPLN | 70.5 (7.1) | 76.8 (6.7) | 74.4 (6.8) | 103.9 (6.4) | 95.2 (7.0) | 95.6 (4.8) |
| LPGM | 5.1 (0.1) | 5.0 (0.1) | 5.3 (0.1) | 13.2 (1.2) | 12.1 (1.0) | 11.9 (1.6) |
| Glasso | 15.6 (0.4) | 15.7 (0.2) | 15.7 (0.3) | 15.6 (0.6) | 15.5 (0.1) | 15.3 (0.7) |
| PPCOR | 0.1 (0.0) | 0.1 (0.0) | 0.1 (0.0) | 0.1 (0.0) | 0.1 (0.0) | 0.1 (0.0) |
| GENIE3 | 1.7 (0.0) | 1.8 (0.0) | 1.9 (0.0) | 1.7 (0.0) | 1.5 (0.0) | 1.6 (0.0) |
| PIDC | **0.1 (0.1)** | **0.0 (0.0)** | **0.0 (0.0)** | **0.0 (0.0)** | **0.0 (0.0)** | **0.0 (0.0)** |
|  | Hub graph | | | | | |
| VMPLN | 62.2 (4.2) | 88.6 (9.7) | 99.5 (11.9) | 83.6 (7.3) | 82.4 (5.3) | 121 (8.3) |
| VPLN | 92.9 (3.9) | 88.1 (6.0) | 91.3 (5.9) | 95.9 (4.0) | 98.5 (4.8) | 175.4 (4.3) |
| LPGM | 4.8 (0.1) | 5.0 (0.1) | 5.0 (0.1) | 16.5 (1.5) | 15.2 (1.6) | 17.5 (1.2) |
| Glasso | 15.6 (0.4) | 15.5 (0.5) | 15.5 (0.4) | 15.5 (0.5) | 15.5 (0.7) | 17.4 (1.4) |
| PPCOR | 0.1 (0.0) | 0.1 (0.0) | 0.1 (0.0) | 0.1 (0.0) | 0.1 (0.0) | 0.1 (0.0) |
| GENIE3 | 1.7 (0.0) | 1.7 (0.0) | 1.7 (0.0) | 1.5 (0.0) | 1.5 (0.0) | 1.7 (0.0) |
| PIDC | **0.0 (0.0)** | **0.0 (0.0)** | **0.0 (0.0)** | **0.0 (0.0)** | **0.0 (0.0)** | **0.0 (0.0)** |
|  | Blocked random graph | | | | | |
| VMPLN | 61.7 (9.6) | 80.9 (12.5) | 109.2 (12.2) | 73.9 (4.0) | 70.8 (11.6) | 100.8 (9.6) |
| VPLN | 74.6 (4.6) | 80.7 (7.4) | 69.6 (4.7) | 99.9 (8.2) | 95.2 (7.8) | 94.0 (5.9) |
| LPGM | 5.4 (0.0) | 5.4 (0.0) | 5.4 (0.0) | 11.5 (0.7) | 12.1 (0.8) | 11.1 (1.2) |
| Glasso | 15.7 (0.3) | 15.7 (0.3) | 15.8 (0.2) | 15.5 (0.7) | 15.7 (0.3) | 15.6 (0.2) |
| PPCOR | 0.1 (0.0) | 0.1 (0.0) | 0.1 (0.0) | 0.1 (0.0) | 0.1 (0.0) | 0.1 (0.0) |
| GENIE3 | 1.9 (0.0) | 1.9 (0.0) | 1.9 (0.0) | 1.5 (0.0) | 1.7 (0.0) | 1.6 (0.0) |
| PIDC | **0.0 (0.0)** | **0.0 (0.0)** | **0.0 (0.0)** | **0.0 (0.0)** | **0.0 (0.0)** | **0.0 (0.0)** |
|  | Scale-free graph | | | | | |
| VMPLN | 56.3 (10.8) | 96.3 (8.4) | 110.2 (9.8) | 76.8 (3.8) | 82.2 (13.1) | 102.3 (9.6) |
| VPLN | 84.5 (4.8) | 86.4 (4.7) | 85.8 (8.9) | 96.8 (5.0) | 103.1 (6.4) | 100.9 (3.4) |
| LPGM | 5.4 (0.1) | 5.3 (0.1) | 5.3 (0.0) | 13.2 (1.1) | 13.4 (1.4) | 12.8 (1.0) |
| Glasso | 15.7 (0.4) | 15.7 (0.2) | 16.0 (0.2) | 15.5 (0.4) | 15.6 (0.4) | 15.5 (0.3) |
| PPCOR | 0.1 (0.0) | 0.1 (0.0) | 0.1 (0.0) | 0.1 (0.0) | 0.1 (0.0) | 0.1 (0.0) |
| GENIE3 | 1.8 (0.0) | 1.8 (0.0) | 1.9 (0.0) | 1.5 (0.0) | 1.6 (0.0) | 1.5 (0.0) |
| PIDC | **0.0 (0.0)** | **0.0 (0.0)** | **0.0 (0.0)** | **0.0 (0.0)** | **0.0 (0.0)** | **0.0 (0.0)** |

**Table. S3: Comparison of GRN inference methods in terms of CPU time (minute) for p = 100 in simulation.** The results are averages over 50 replicates with standard deviations in brackets.

| Dimension | p=300 | | | p=300 | | |
| --- | --- | --- | --- | --- | --- | --- |
| Dropout | Low | | | High | | |
|  | Random graph | | | | | |
| Mixing degree | Low | Middle | High | Low | Middle | High |
| VMPLN | 276.4 (39.6) | 381.1 (89.9) | 498.5 (13.7) | 363.7 (54.5) | 417.2 (30.5) | 539.3 (38.3) |
| VPLN | 392.1 (25.4) | 398.9 (15.8) | 411.9 (9.7) | 403.7 (24.1) | 384.7 (19.7) | 428.8 (14.8) |
| LPGM | 41.5 (0.3) | 42.6 (0.7) | 45.1 (1.1) | 139.0 (4.7) | 135.8 (7.4) | 138.4 (6.0) |
| Glasso | 46.8 (0.5) | 46.0 (1.0) | 45.7 (0.8) | 44.4 (0.5) | 44.1 (0.6) | 44.3 (0.7) |
| PPCOR | **0.3 (0.0)** | **0.4 (0.0)** | **0.3 (0.0)** | **0.4 (0.0)** | **0.4 (0.0)** | **0.4 (0.0)** |
| GENIE3 | 9.1 (0.0) | 9.3 (0.1) | 9.3 (0.1) | 8.0 (0.0) | 8.1 (0.0) | 8.2 (0.0) |
| PIDC | 0.6 (0.0) | 0.7 (0.0) | 0.6 (0.0) | 0.7 (0.0) | 0.7 (0.0) | 0.7 (0.0) |
|  | Hub graph | | | | | |
| VMPLN | 255.6 (14.8) | 348.9 (59.0) | 430.6 (15.0) | 432.0 (56.6) | 469.7 (67.6) | 622.3 (39.0) |
| VPLN | 385.9 (21.8) | 393.1 (15.8) | 396.2 (16.6) | 385.9 (16.8) | 367.7 (15.9) | 392.4 (20.8) |
| LPGM | 41.7 (0.6) | 42.5 (0.9) | 45.4 (0.9) | 152.6 (6.0) | 152.2 (9.6) | 157.0 (6.8) |
| Glasso | 47.3 (0.2) | 46.8 (1.7) | 46.3 (0.4) | 44.5 (0.6) | 44.1 (0.6) | 44.5 (0.3) |
| PPCOR | **0.4 (0.0)** | **0.3 (0.0)** | **0.4 (0.0)** | **0.4 (0.0)** | **0.4 (0.0)** | **0.3 (0.0)** |
| GENIE3 | 8.8 (0.0) | 8.9 (0.1) | 9.0 (0.1) | 7.6 (0.0) | 7.7 (0.1) | 7.7 (0.1) |
| PIDC | 0.7 (0.0) | 0.7 (0.0) | 0.7 (0.0) | 0.7 (0.0) | 0.7 (0.0) | 0.7 (0.0) |
|  | Blocked random graph | | | | | |
| VMPLN | 264.6 (23.3) | 279.3 (61.8) | 424.2 (31.9) | 398.8 (37.5) | 387.2 (49.4) | 515.6 (40.6) |
| VPLN | 405.8 (27.0) | 394.7 (28.7) | 420.9 (14.8) | 424.3 (11.1) | 404.9 (7.3) | 409.6 (14.2) |
| LPGM | 41.8 (0.3) | 42.4 (1.4) | 45.0 (1.1) | 140.7 (6.8) | 145.3 (10.4) | 140.0 (5.0) |
| Glasso | 46.4 (0.7) | 46.8 (1.1) | 46.1 (0.5) | 44.5 (0.5) | 44.0 (0.5) | 43.7 (0.6) |
| PPCOR | **0.4 (0.0)** | **0.3 (0.0)** | **0.4 (0.0)** | **0.4 (0.0)** | **0.4 (0.0)** | **0.4 (0.0)** |
| GENIE3 | 9.1 (0.0) | 9.2 (0.2) | 9.4 (0.1) | 8.0 (0.0) | 8.1 (0.1) | 7.7 (0.1) |
| PIDC | 0.7 (0.0) | 0.7 (0.0) | 0.7 (0.0) | 0.7 (0.0) | 0.7 (0.0) | 0.7 (0.0) |
|  | Scale-free graph | | | | | |
| VMPLN | 231.4 (46.4) | 310.7 (47.9) | 461.3 (17.5) | 427.3 (39.0) | 417.4 (34.8) | 496.1 (40.3) |
| VPLN | 382.3 (21.2) | 383.7 (23.3) | 435.1 (18.0) | 399.0 (14.9) | 417.0 (22.1) | 422.7 (13.6) |
| LPGM | 44.2 (0.9) | 41.0 (1.4) | 44.8 (1.1) | 137.0 (9.9) | 146.9 (9.1) | 139.7 (6.4) |
| Glasso | 45.2 (0.7) | 44.4 (0.7) | 44.7 (0.6) | 44.0 (0.5) | 44.0 (0.5) | 44.0 (0.5) |
| PPCOR | **0.3 (0.0)** | **0.3 (0.0)** | **0.3 (0.0)** | **0.3 (0.0)** | **0.4 (0.0)** | **0.4 (0.0)** |
| GENIE3 | 9.2 (0.1) | 8.8 (0.1) | 9.4 (0.1) | 8.1 (0.0) | 8.2 (0.1) | 8.2 (0.1) |
| PIDC | 0.7 (0.0) | 0.7 (0.0) | 0.7 (0.0) | 0.7 (0.1) | 0.7 (0.0) | 0.7 (0.0) |

**Table. S4: Comparison of GRN inference methods in terms of CPU time (minute) for p = 300 in simulation.** The results are averages over 50 replicates with standard deviations in brackets.

**S2 Supplementary: Benchmarking on scRNA-seq Data**

**S2.1 The Description of Benchmarking Datasets**

| Dataset | Cell type | Cells number |
| --- | --- | --- |
| Kang data ([2](#_ENREF_2)) | CD14+ monocytes | 2147 |
|  | CD16+ monocytes | 537 |
|  | Dendritic cells | 214 |
|  | CD4+ naive T cells | 1526 |
|  | CD4+ memory T cells | 903 |
|  | CD8+ T cells | 462 |
|  | Natural killer cells | 321 |
|  | Activated T cells | 333 |
|  | B cells | 571 |
|  | Activated B cells | 203 |
|  | Megakaryocytes | 121 |
|  | Plasmacytoid dendritic cells | 81 |
|  | Erythrocytes | 32 |
| Zheng data ([3](#_ENREF_3)) | FCGR3A+ monocytes | 355 |
|  | CD14+ monocytes | 2176 |
|  | Natural killer cells | 290 |
|  | CD8+ T cells | 1066 |
|  | CD4+ T cells | 2183 |
|  | B cells | 1172 |
|  | Hematopoietic stem cell | 7 |
|  | Megakaryocyte | 57 |
|  | Plasmacytoid dendritic cell | 72 |

**Table. S5: The cell numbers of each cell type in the two benchmarking datasets.**


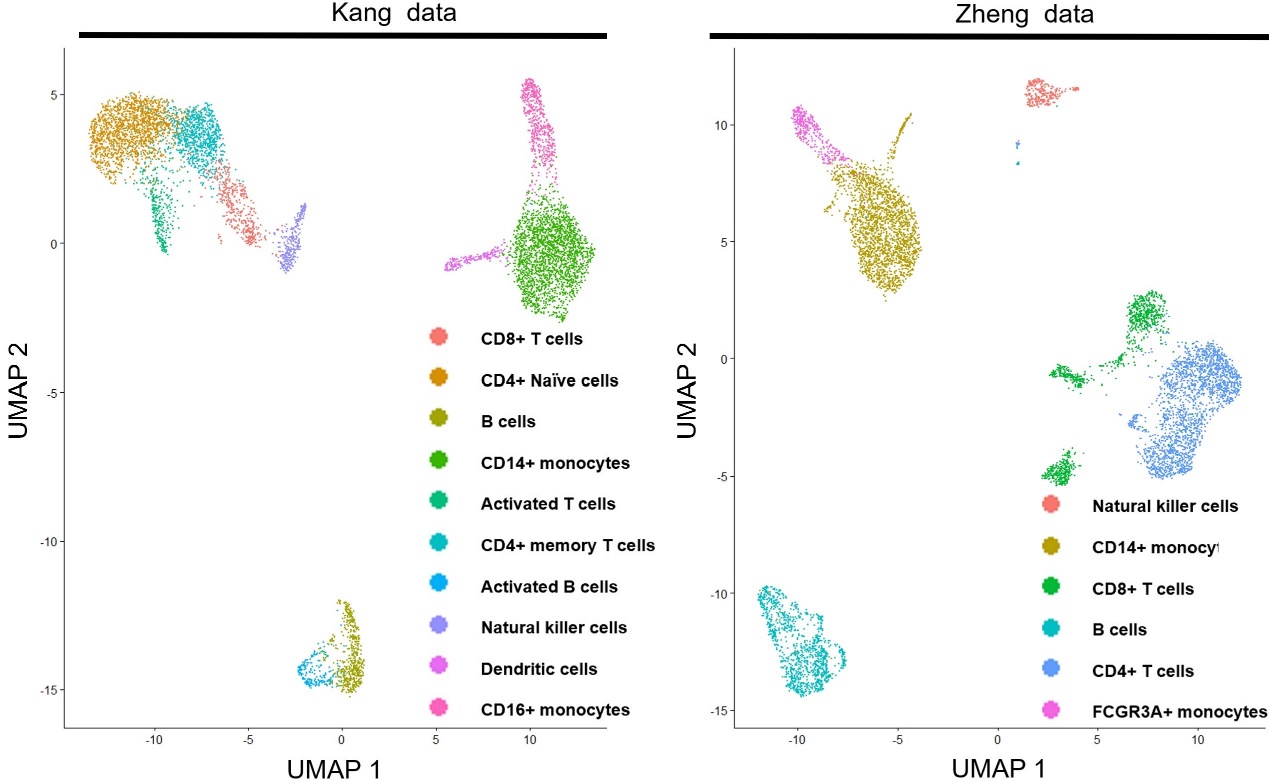


**Fig. S2: The uniform manifold approximation and projection (UMAP) embedding of two benchmarking datasets** ([2](#_ENREF_2), [3](#_ENREF_3)). The cells are colored by annotated cell types.

**S2.2 The Used Public Gene Regulatory Databases**

We use the following public gene regulatory databases for benchmarking analysis as well as providing a prior set of potential edges for gene regulatory network inference in the SARS-COV-2 dataset.

| Type | Source | Link |
| --- | --- | --- |
| PPI databases | STRING ([4](#_ENREF_4)) | https://string-db.org/ |
|  | HumanTFDB ([5](#_ENREF_5)) | http://bioinfo.life.hust.edu.cn/HumanTFDB#!/ |
| ChIP-seq databases | hTFtarget ([6](#_ENREF_6)) | http://bioinfo.life.hust.edu.cn/hTFtarget#!/ |
|  | ChEA ([7](#_ENREF_7)) | https://maayanlab.cloud/Harmonizome/dataset/CHEA+Transcription+Factor+Targets |
|  | ChIP-Atlas ([8](#_ENREF_8)) | https://chip-atlas.org/peak browser |
|  | ChIPBase ([9](#_ENREF_9)) | https://rna.sysu.edu.cn/chipbase/ |
|  | ESCAPE ([10](#_ENREF_10)) | http://www.maayanlab.net/ESCAPE/download.php |
| Integrated databases | TRRUST ([11](#_ENREF_11)) | https://www.grnpedia.org/trrust/ |
|  | RegNetwork ([12](#_ENREF_12)) | http://www.regnetworkweb.org/ |

**Table. S6: The used public GRN databases.**

**S2.3 Silver Standard Construction for Benchmarking on scRNA-seq Data**

The Kang data consists of two batches, the interferon Beta 1 (IFNB1)-stimulated and control groups. The Zheng data also consists of two batches, which are respectively sequenced by $3^{'}$ and $5^{'}$ scRNA-seq technologies. Silver standards are constructed using the IFNB1-stimulated group and the $3^{'}$ batch for the Kang data and Zheng data, respectively. The gene pairs that occur in the public gene regulatory network databases (see Supplementary Table. S6) are taken as potential regulatory relationships. Each of these potential regulatory relationships involves at least one transcription factor. Then, for each cell type in the construction batch, we calculate the Spearman's $\rho$ correlation between the gene pairs having potential regulatory relationships. If a gene pair has a significant Spearman's $\rho$ correlation, we consider the gene pair having a true regulatory relationship and add the edge to the silver standard edge set of the cell type.

**S2.4** **Additional Result of Benchmarking on scRNA-seq Data**

**
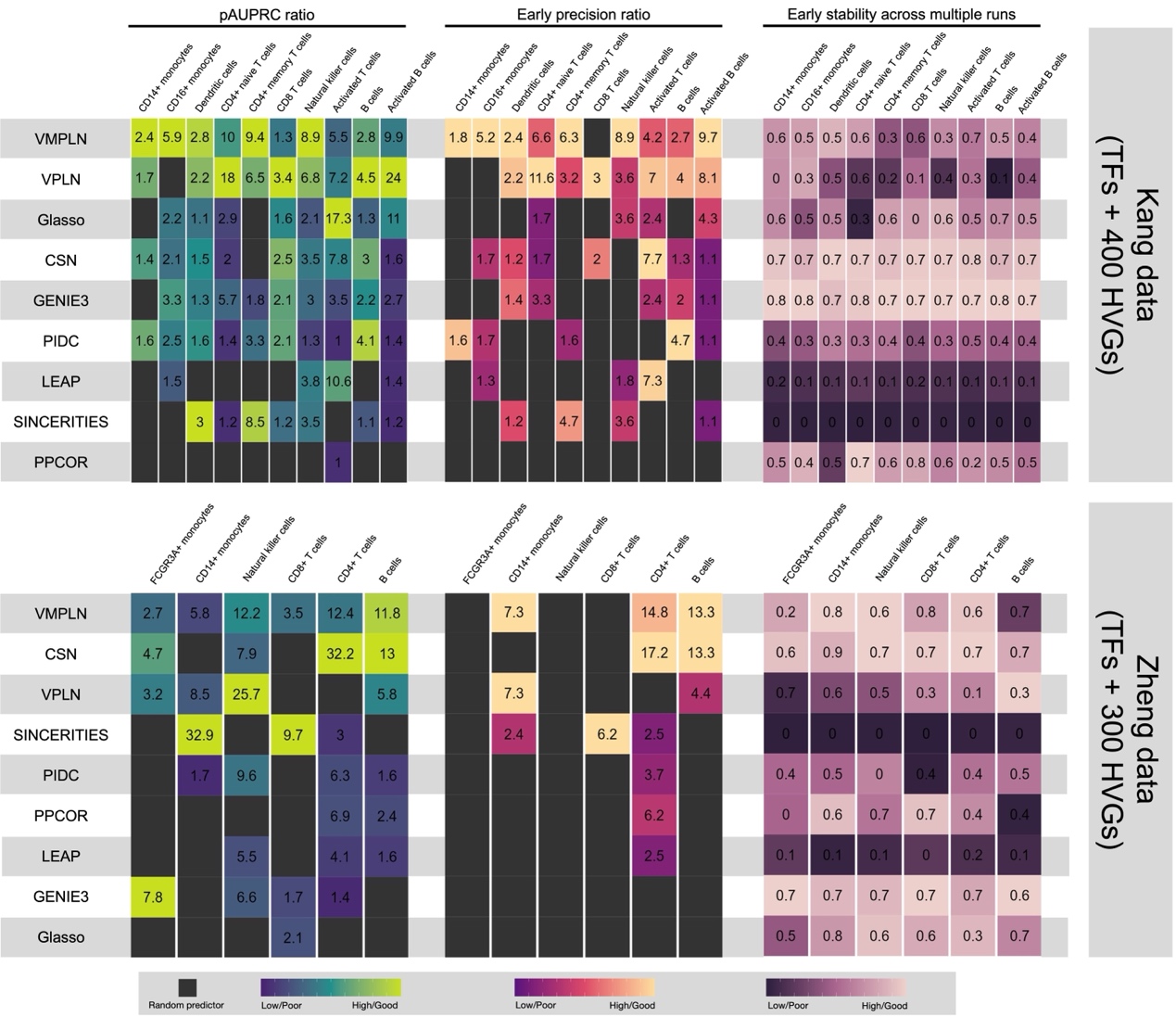
**

**Fig. S3 The performance of the network inference algorithms in two evaluation scRNA-seq datasets using TFs and 300 and 400 most highly variable genes.** The colors represent the scaled values of these metrics within each cell type, and the actual values are marked in the boxes. Black color in the boxes: the random predictor performs better.

**S3 Supplementary: Application to scRNA-seq Data from COVID-19 Patients**

**S3.1 The Description of SARS-COV-2 Dataset**

| COVID-19 severity | Patient ID | Group 1 | Group 2 | Group 3 | Group 4 |
| --- | --- | --- | --- | --- | --- |
| Moderate | M1 | 102 | 1183 | 365 | 470 |
|  | M2 | 62 | 963 | 397 | 702 |
| Severe | S1 | 1884 | 4104 | 2378 | 1223 |
|  | S2 | 3443 | 6854 | 1452 | 720 |
|  | S3 | 175 | 231 | 112 | 117 |
|  | S4 | 396 | 374 | 212 | 103 |
|  | S5 | 243 | 529 | 152 | 124 |
|  | S6 | 194 | 540 | 101 | 175 |

**Table. S7: The cell number of each cell type in each patient in the COVID-19 dataset** ([13](#_ENREF_13)).

**
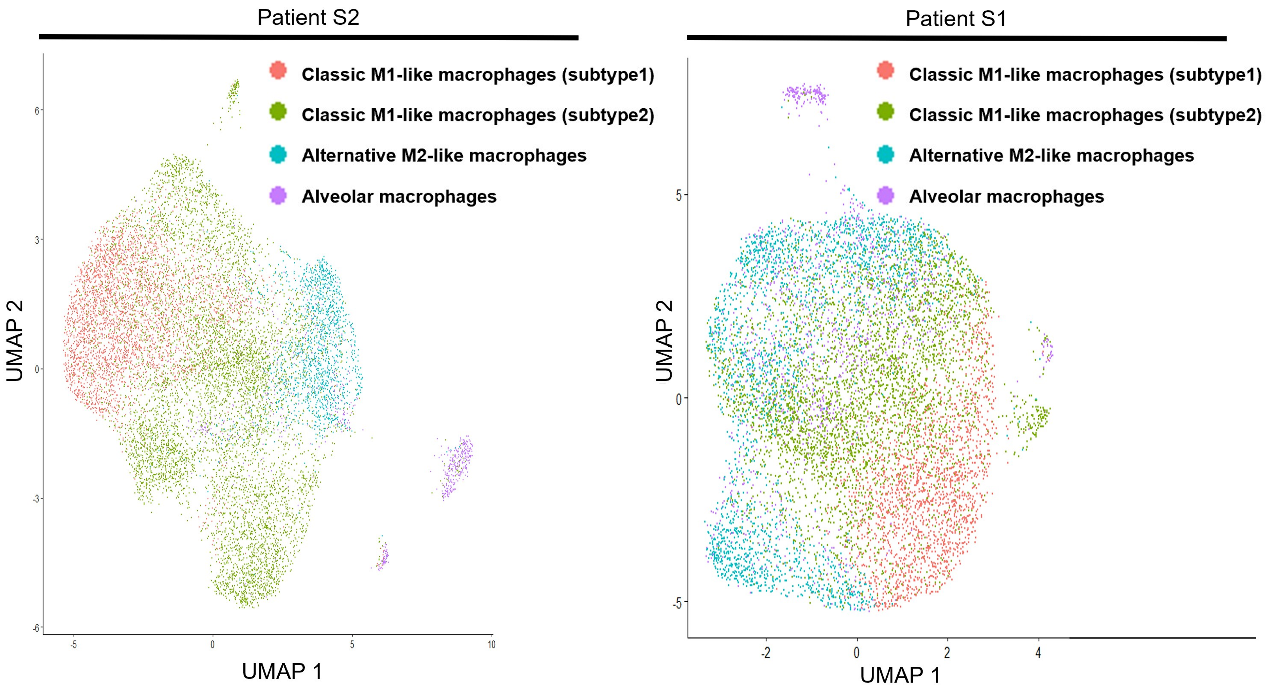
**

**Fig. S4: The uniform manifold approximation and projection (UMAP) embedding of patient S1 and S2 in SARS-CoV-2 dataset** ([13](#_ENREF_13)). The cells are colored by annotated cell types.

**S3.2 Additional Details of Gene Regulatory Network Inference of SARS-COV-2 Dataset**

We select the top 2000 highly variable genes for each patient and use the union of the highly variable genes from all patients for VMPLN analysis. The gene set of interest for gene regulatory network inference is selected as the overall top 1000 highly variable genes as defined by Seurat ([14](#_ENREF_14)). The edges that do not appear in the public gene regulatory network databases listed in Supplementary Table. S6 are set to 0. We only focus on the gene regulatory network among genes in the gene set of interest for gene regulatory network inference. We perform VMPLN analysis for each patient separately and select the parameters such that the density of the estimated networks (i.e., the number of inferred edges divided by the number of edges in the prior gene regulatory network set) is about 5%. Then, for each macrophage group, we weighted the average of the estimated partial correlations from each moderate patient with the number of cells as a weight to get the gene regulatory network under the moderate condition. Similarly, we obtain the gene regulatory network for every macrophage group under severe condition.


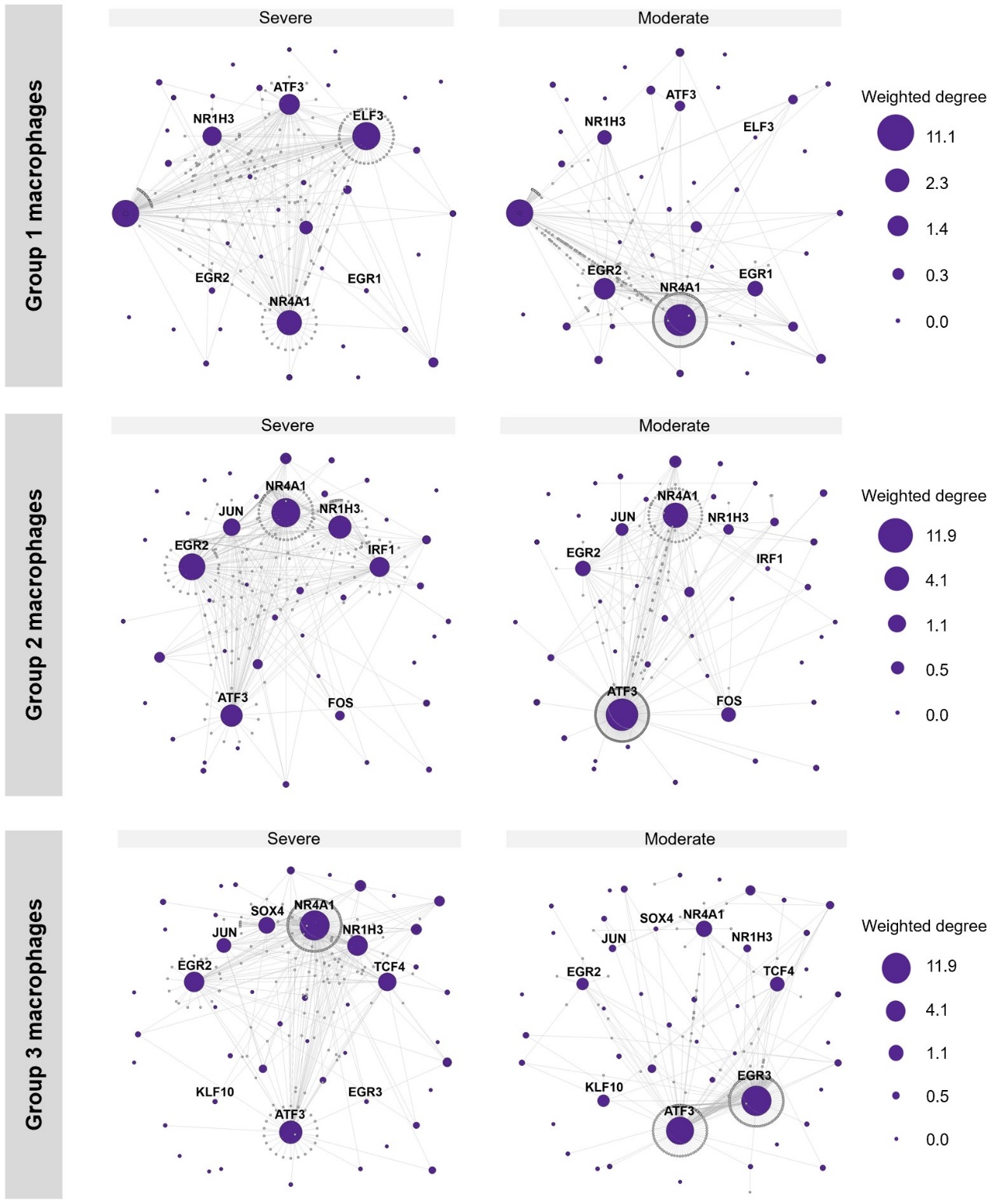


**Fig. S5: The inferred gene regulatory networks (similar to Fig. 5a) of Group1, Group2, and Group3 macrophages in severe and moderate patients.**


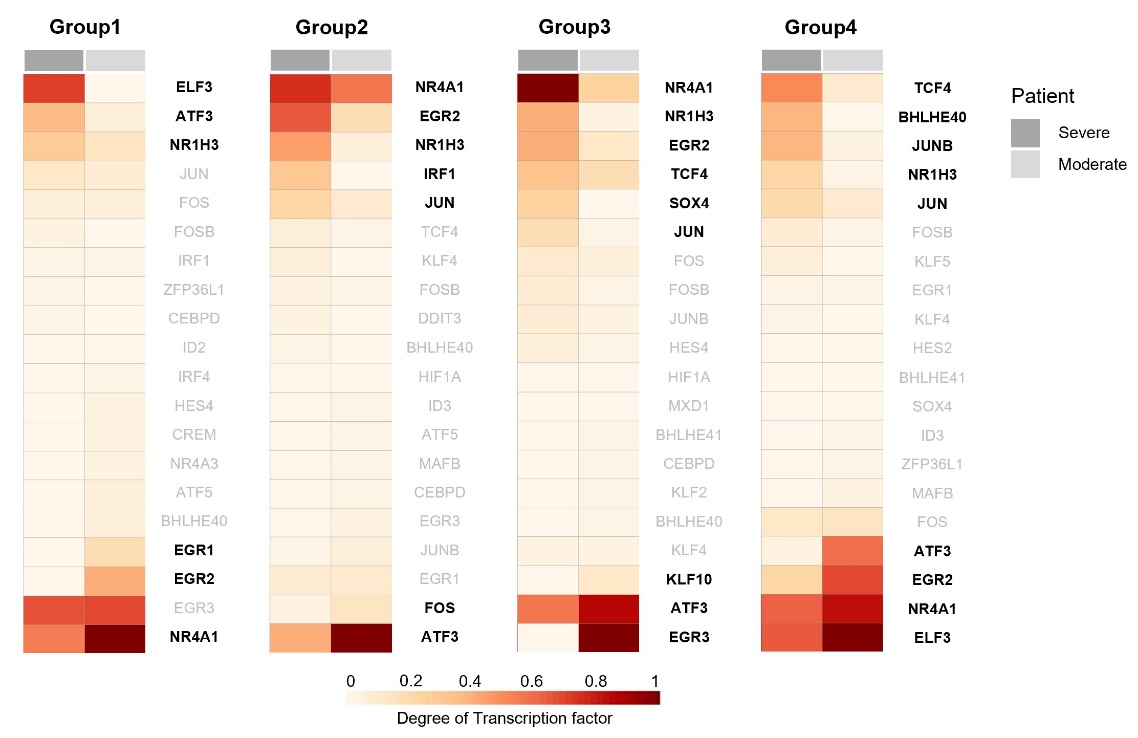


**Fig. S6: The scaled node degree of TFs of four macrophage groups between severe patients and moderate patients.** The node degree is calculated based on the absolute partial correlation matrix induced by the estimated precision matrix. Black in bold represents TFs that exhibit a large degree difference between the GRNs in the moderate and severe patients (greater than 0.2).


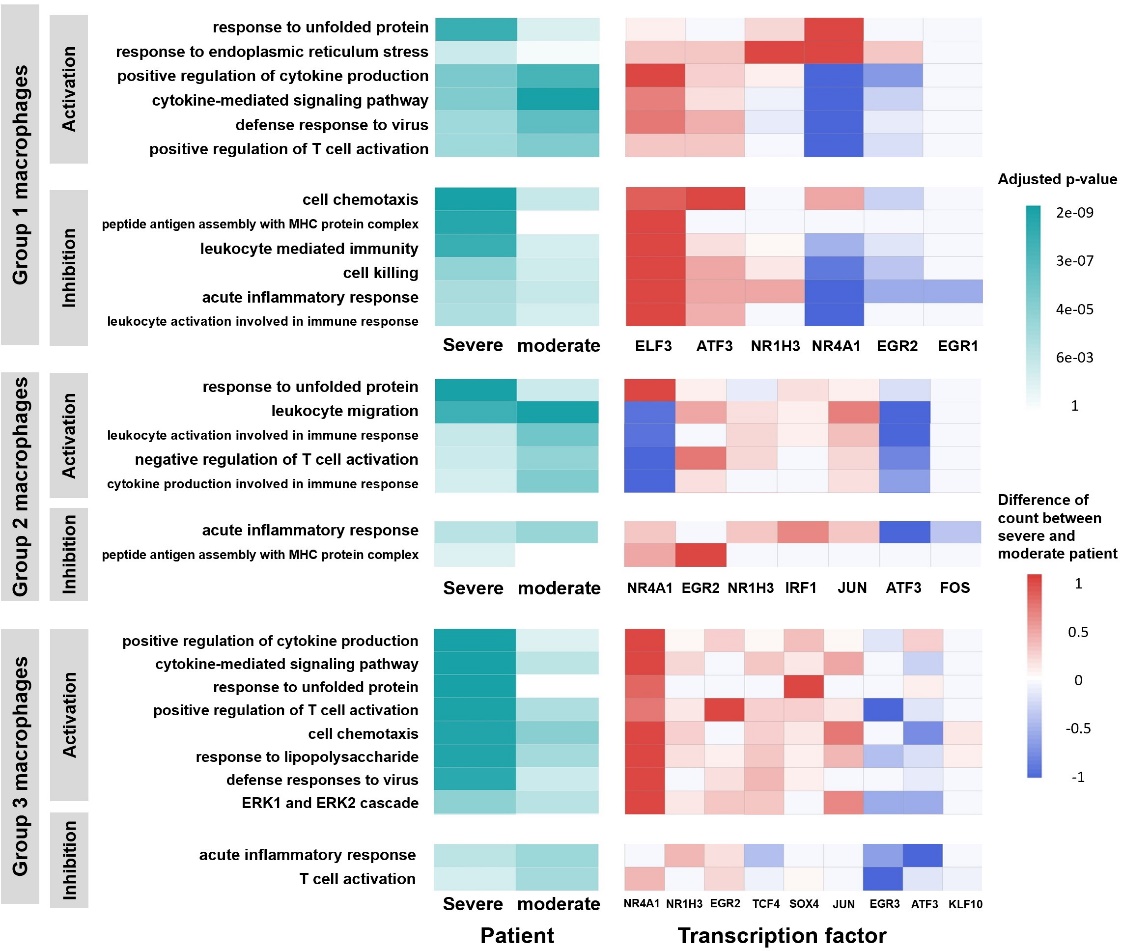


**Fig. S7: GO enrichment analysis of TFs' target genes (similar to Fig. 5b) for Group1, Group2, and Group3 macrophages.**

References

1. Hartigan JA, Wong MA. Algorithm AS 136: A k-means clustering algorithm. Journal of the royal statistical society series c (applied statistics). 1979;28(1):100-8.

2. Kang HM, Subramaniam M, Targ S, Nguyen M, Maliskova L, McCarthy E, et al. Multiplexed droplet single-cell RNA-sequencing using natural genetic variation. Nature biotechnology. 2018;36(1):89-94.

3. Zheng G, Terry J, Belgrader P, Ryvkin P, Bent Z, Wilson R, et al. Massively parallel digital transcriptional profiling of single cells. Nat Commun 8: 14049. Data Set5 Putative transcription factors binding motifs identified for genes in trans‐eQTL (expression quantitative trait loci) hotspots Data Set6 Putative master regulators in the trans‐eQTL (expression quantitative trait loci) hotspots Figure S. 2017;1.

4. Szklarczyk D, Gable AL, Lyon D, Junge A, Wyder S, Huerta-Cepas J, et al. STRING v11: protein–protein association networks with increased coverage, supporting functional discovery in genome-wide experimental datasets. Nucleic acids research. 2019;47(D1):D607-D13.

5. Hu H, Miao Y-R, Jia L-H, Yu Q-Y, Zhang Q, Guo A-Y. AnimalTFDB 3.0: a comprehensive resource for annotation and prediction of animal transcription factors. Nucleic acids research. 2019;47(D1):D33-D8.

6. Zhang Q, Liu W, Zhang H-M, Xie G-Y, Miao Y-R, Xia M, Guo A-Y. hTFtarget: a comprehensive database for regulations of human transcription factors and their targets. Genomics, proteomics & bioinformatics. 2020;18(2):120-8.

7. Lachmann A, Xu H, Krishnan J, Berger SI, Mazloom AR, Ma'ayan A. ChEA: transcription factor regulation inferred from integrating genome-wide ChIP-X experiments. Bioinformatics. 2010;26(19):2438-44.

8. Oki S, Ohta T, Shioi G, Hatanaka H, Ogasawara O, Okuda Y, et al. Ch IP‐Atlas: a data‐mining suite powered by full integration of public Ch IP‐seq data. EMBO reports. 2018;19(12):e46255.

9. Zhou K-R, Liu S, Sun W-J, Zheng L-L, Zhou H, Yang J-H, Qu L-H. ChIPBase v2. 0: decoding transcriptional regulatory networks of non-coding RNAs and protein-coding genes from ChIP-seq data. Nucleic acids research. 2016:gkw965.

10. Xu H, Baroukh C, Dannenfelser R, Chen EY, Tan CM, Kou Y, et al. ESCAPE: database for integrating high-content published data collected from human and mouse embryonic stem cells. Database. 2013;2013:bat045.

11. Han H, Cho J-W, Lee S, Yun A, Kim H, Bae D, et al. TRRUST v2: an expanded reference database of human and mouse transcriptional regulatory interactions. Nucleic acids research. 2018;46(D1):D380-D6.

12. Liu Z-P, Wu C, Miao H, Wu H. RegNetwork: an integrated database of transcriptional and post-transcriptional regulatory networks in human and mouse. Database. 2015;2015:bav095.

13. Liao M, Liu Y, Yuan J, Wen Y, Xu G, Zhao J, et al. Single-cell landscape of bronchoalveolar immune cells in patients with COVID-19. Nature medicine. 2020;26(6):842-4.

14. Stuart T, Butler A, Hoffman P, Hafemeister C, Papalexi E, Mauck WM, et al. Comprehensive integration of single-cell data. Cell. 2019;177(7):1888-902. e21.
